# Chemical biology and medicinal chemistry of RNA methyltransferases

**DOI:** 10.1093/nar/gkac224

**Published:** 2022-04-12

**Authors:** Tim R Fischer, Laurenz Meidner, Marvin Schwickert, Marlies Weber, Robert A Zimmermann, Christian Kersten, Tanja Schirmeister, Mark Helm

**Affiliations:** Institute of Pharmaceutical and Biomedical Sciences, Johannes Gutenberg-University Mainz, Staudingerweg 5, 55128Mainz, Germany; Institute of Pharmaceutical and Biomedical Sciences, Johannes Gutenberg-University Mainz, Staudingerweg 5, 55128Mainz, Germany; Institute of Pharmaceutical and Biomedical Sciences, Johannes Gutenberg-University Mainz, Staudingerweg 5, 55128Mainz, Germany; Institute of Pharmaceutical and Biomedical Sciences, Johannes Gutenberg-University Mainz, Staudingerweg 5, 55128Mainz, Germany; Institute of Pharmaceutical and Biomedical Sciences, Johannes Gutenberg-University Mainz, Staudingerweg 5, 55128Mainz, Germany; Institute of Pharmaceutical and Biomedical Sciences, Johannes Gutenberg-University Mainz, Staudingerweg 5, 55128Mainz, Germany; Institute of Pharmaceutical and Biomedical Sciences, Johannes Gutenberg-University Mainz, Staudingerweg 5, 55128Mainz, Germany; Institute of Pharmaceutical and Biomedical Sciences, Johannes Gutenberg-University Mainz, Staudingerweg 5, 55128Mainz, Germany

## Abstract

RNA methyltransferases (MTases) are ubiquitous enzymes whose hitherto low profile in medicinal chemistry, contrasts with the surging interest in RNA methylation, the arguably most important aspect of the new field of epitranscriptomics. As MTases become validated as drug targets in all major fields of biomedicine, the development of small molecule compounds as tools and inhibitors is picking up considerable momentum, in academia as well as in biotech. Here we discuss the development of small molecules for two related aspects of chemical biology. Firstly, derivates of the ubiquitous cofactor *S*-adenosyl-l-methionine (SAM) are being developed as bioconjugation tools for targeted transfer of functional groups and labels to increasingly visible targets. Secondly, SAM-derived compounds are being investigated for their ability to act as inhibitors of RNA MTases. Drug development is moving from derivatives of cosubstrates towards higher generation compounds that may address allosteric sites in addition to the catalytic centre. Progress in assay development and screening techniques from medicinal chemistry have led to recent breakthroughs, e.g. in addressing human enzymes targeted for their role in cancer. Spurred by the current pandemic, new inhibitors against coronaviral MTases have emerged at a spectacular rate, including a repurposed drug which is now in clinical trial.

## INTRODUCTION

Concomitant with a surge in research and publication activity about a decade ago, the field of RNA modification was dubbed ‘epitranscriptomics’ (1,2). This term was coined in analogy to the field of epigenetics to imply the transport of information that is not encoded in the sequence of nucleic acids. The single most important type of such information is conveyed by RNA methylation, a reaction catalyzed by RNA methyltransferases (MTases), frequently addressed as ‘writers’ of such information. MTases are a major enzyme class that has, until recently, received some attention in chemical biology, but less so in medicinal chemistry.

Especially in comparison to other enzymes catalyzing group transfer reactions, such as kinases (3–5), literature on MTases in general (6,7), and on RNA MTases in particular, is surprisingly scarce. Because involvement of RNA MTases in numerous pathologies has become increasingly apparent in recent years, they are now being targeted for small molecule drug development, as evidenced by the appearance of a number of start-up biotech companies (8–11). Development of resulting compounds as antibiotics or anti-cancer drugs is considered highly promising.

Because this recent development is liable to entail increased activity in the field of medicinal chemistry, we decided to collect and compile pertinent literature and to summarize the state of science. In doing so, we acknowledge that, in addition to a relatively low number of small molecules available in the literature for modulating RNA MTase activity, there is a related field that provides valuable insight in terms of structure–activity relationships (SAR). Interestingly, the use of functional *S*-adenosyl-l-methionine (SAM) analogues for group transfer was first developed with DNA, before being applied to RNA as well, therein recapitulating the dynamic research interest in the fields of epigenetics and epitranscriptomics.

The vast majority of MTases utilizes SAM as cofactor (12) with few exceptions using *N*^5^,*N*^10^-methylenetetrahydrofolate (MTHF) (13–16). SAM MTases, which are found in all three domains of life, have been categorized into 9 classes of which class I to V have already been reviewed elsewhere (12,17–19). Among them class I the so called Rossmann-fold MTases are the most frequent ones (12,17,19,20), whereas the structure of Class II, III and V is determined by their specific function (21–23). The classification is based on the enzymes’ characteristic structural fold and is independent from its phylogenetic content (24–26). Noteworthy, only classes, I, IV, VII, VIII and IX have been described to include RNA MTases. A brief overview is given in Table 1 including exemplary crystal structures and references for further information.

**Table 1.** Overview of MTase classification

Name	Structure	Exemplary crystal structure
**Class I Rossmann-Fold MTases** (264,286–288)	Seven β-sheets flanked by three α-helices on each side forming an αβα-sandwich	nsp10-nsp16 methyltransferase complex SARS-CoV-2 PDB 6YZ1 (264)
**ClassII**(21)	Eight central β-sheets framed by several α-helices at both ends including a characteristic central, antiparallel β-sheet	MetH methionine synthase *E. coli K12* PDB 1MSK (21)
**Class III** (22)	Two αβ-clusters each containing five central β-sheets surrounded by four α-helices	CbiF cobalt-precorrin-4 transmethylase *Priestia megaterium* PDB 1CBF (22)
**Class IV SPOUT (SpoU-TrmD) MTases** (26,289)	Characteristic knot structure at the C terminus; six-stranded parallel β-sheet surrounded by seven α-helices	RlmB 2′-*O*-methyltransferase *E. coli* PDB: 1GZ0 (289)
**Class V SET-domain containing proteins** (23,290)	Combination of three β-sheet formations each consisting of three β-strands surrounding the remarkable ‘pseudoknot’ at the C terminus	SET7/9 lysine methyltransferase *H. sapiens* PDB: 1MT6 (23)
**Class VI Transmembrane MTases** (291,292)	Five transmembrane α-helices and a cofactor-binding pocket enclosed within a highly conserved C-terminal catalytic subdomain comprising an α-helix.	Isoprenylcysteine carboxyl methyltransferase (ICMT), *Methanosarcina acetivorans* PDB: 4A2N (291)
**ClassVII TIM barrel MTases** (293,294)	Partial (α/β)_6_ TIM barrel or full TIM barrel in some cases: a β-sheet is anchored inside by peripheral α-helices, on top the [4Fe-4S] cluster.	MiaB radical methylthiotransferase *Bacteroides uniformis* PDB: 7MJV (294)
**ClassVIII**(295,296)	Six-stranded anti-parallel β-barrel, β-sheets are connected by numerous extended loops.	TrmO/YaeB tRNA methyltransferase *Archaeoglobus fulgidus* PDB: 2NV4 (295,296)
**Class IX** (297)	Two four-stranded antiparallel β-sheets forming a concave surface with five α-helices at the reverse side of the protein.	SsTaw3 tRNA-yW N-4 Methyltransferases *Saccharolobus solfataricus* PDB: 1TLJ (297)

The table includes information about characteristic structural features and examples of enzyme structures.

On the contrary, MTHF-dependent MTases have not been put into a generally accepted classification system, even though they too, are found in all three domains of life (27), methylate a broad collection of substrates, like small molecules, RNA and proteins (28–30). They include clinically relevant drug targets such as the thymidylate synthase, the target of the anti-cancer drug 5-fluorouracil (5-FU) (31,32).

**Figure 1.**
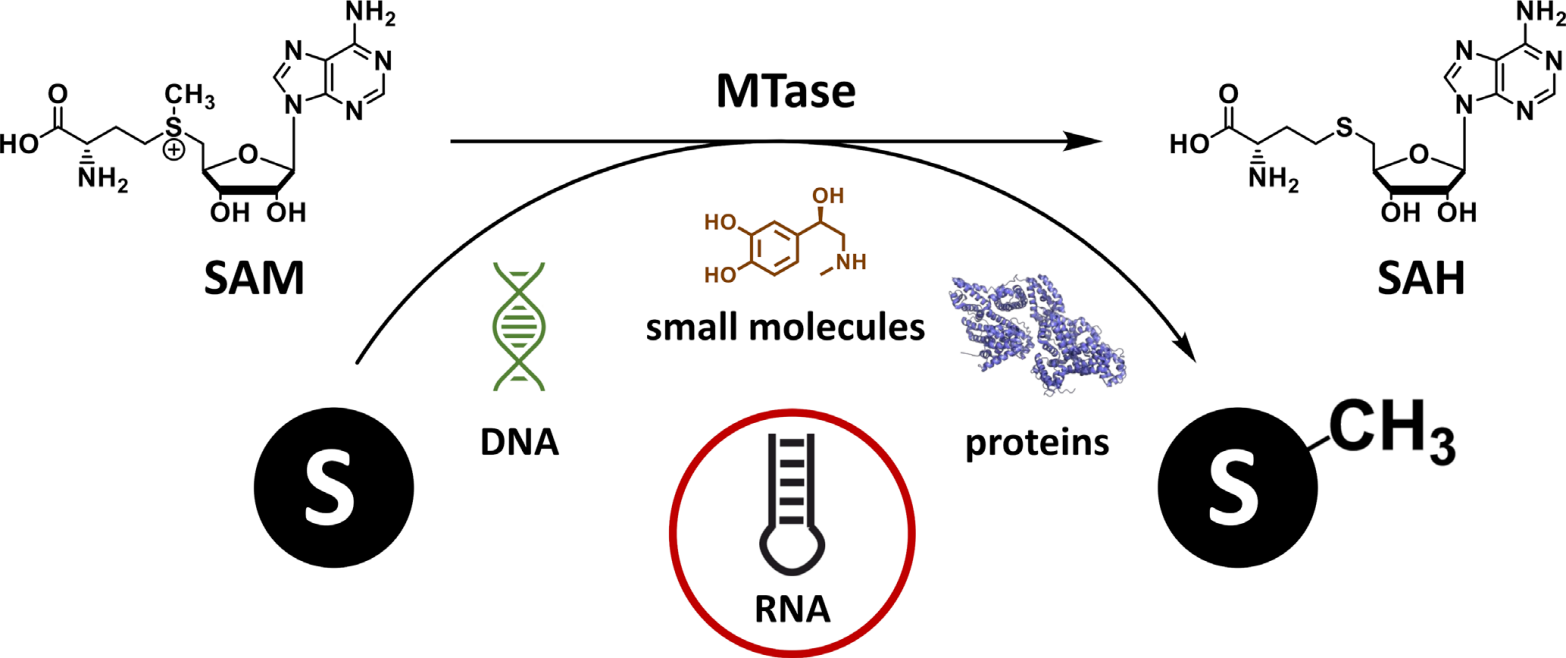
Methylation reactions performed by MTases. Methyl groups are transferred to DNA, small molecules, proteins, or RNA, leading to the formation of SAH as byproduct. Protein structure created with PyMOL (Schrödinger LLC (2010) The PyMOL Molecular Graphics System, Version 1.3.). PDB ID used: 6PWW (280).

SAM dependent MTases are known to methylate a wide variety of biomolecules, e.g. DNA (33), RNA (34), proteins (6) and small molecules (35) as illustrated in Figure 1, yet we will restrict our overview to nucleic acids, with a clear focus on RNA MTases.

## MTASES FOR GROUP TRANSFER REACTIONS ONTO NUCLEIC ACIDS

Among the first attempts to exploit or manipulate the transfer activity of MTases was the use of SAM analogues with moderately modified side chains. Early work found that, although transferring saturated chains longer than methylgroups was possible, transfer ability decreased with increasing chain length (36–38), suggesting a dead end. After a gap in activity, several new molecular concepts were developed to increase side chain transferability for applications of site-specific labelling conferred by the high specificity of the enzymes.

**Figure 2.**
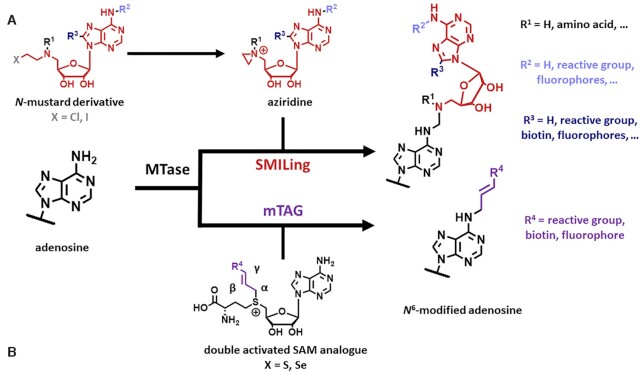
Concepts for group transfer by MTases. (**A**) Reaction scheme for SMILing (sequence-specific methyltransferase-induced labelling) using aziridine derivatives of SAM, which can be produced *in situ* from the corresponding *N*-mustard derivatives. (**B**) Reaction scheme for mTAG using SAM derivatives with β,γ-unsaturated side chains.

The first MTase mediated labelling was described for DNA. A plain aziridine derivative (structure shown in Figure 2A) of adenosine was transferred to the DNA backbone with the enzyme MtaqI (39). Furthermore, so called *N-*mustard derivatives for the *in situ* generation of aziridines have been developed (40). Using more complex molecules, this technique was applied to transferring reactive groups (40–44) and biotin residues (45) for subsequent labelling of the DNA (Figure 2A). Further development led to an approach for fluorescent labelling of DNA in one step (46).

Because the employed enzymes originated from bacterial restriction/modification systems with known sequence specificity for restriction sites, targeting of the labels was straightforward. The aziridine based **s**equence-specific **m**ethyltransferase-**i**nduced **l**abelling was hence dubbed ‘SMILing’ (47). Because the high affinity of the MTases for the reaction product impeded dissociation and therefore multiple turnover (48), efficient labelling reactions required stoichiometric amounts of enzyme (39,49).

To avoid this problem, the field turned back to the transfer of SAM analogue side chains, rather than of the entire cofactor. The activation of the transferable alkyl chain resulting from a central trivalent sulfur atom had shown to be adequate for methylgroups, but insufficient for transfer of longer saturated alkyl chains (*vide infra*). Two approaches to improve this activation were developed. On one hand, the sulfur was replaced with higher chalcogenes (i.e. selenium and tellurium), on the other hand, unsaturated systems were introduced to stabilize the transition state of the transfer reaction.

**Figure 3.**
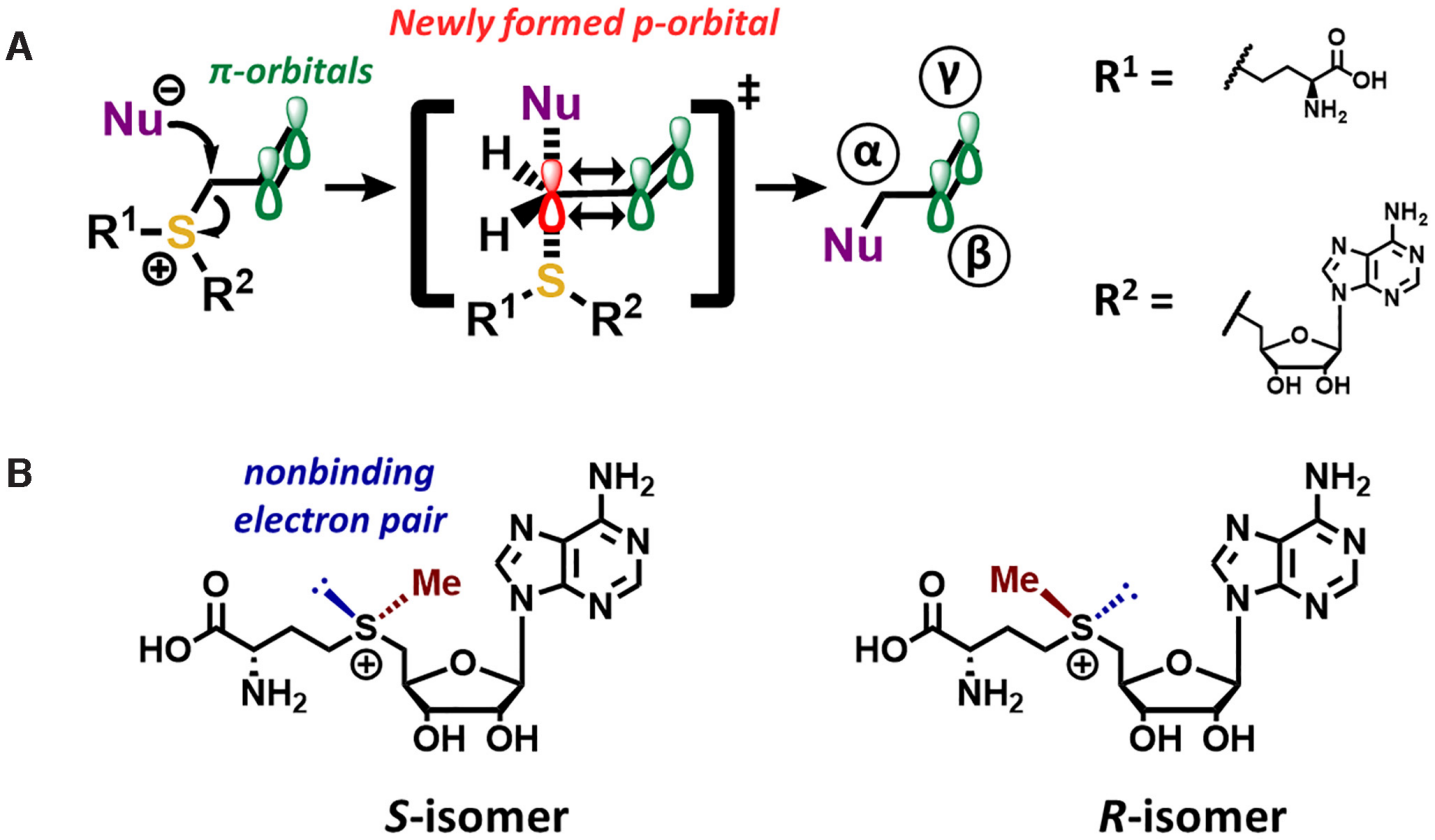
Chemical concepts in biological chemistry of SAM derivatives. (**A**) The β,γ-double bonds of SAM analogues used in mTAG stabilize the transition state formed during nucleophilic attacks in which a nucleophile (Nu^–^, marked in purple) attacks the side chain's α carbon atom. The attack resolves the sp^3^-hybridisation and a sp^2^-like hybridization state with a new p-orbital is formed (marked in red). Here, the newly generated p-orbital now overlaps with π-orbitals of the double bond (marked in green). This stabilization of the transition state decreases the activation energy of the reaction and accelerates it (38,52,53). (**B**) The central sulfur atom of SAH is connecting two parts of the molecule committing two electrons into two single bonds. Like other elements of the sixth group of the periodic table (chalcogens) this leaves four of the six valence electrons forming two nonbinding (also known as ‘lone’) electron pairs. Upon methylation of SAH, one of these nonbinding electron pairs is shared with the methyl group (marked in red) resulting in an electron deficient positively charged sulfur. In higher chalcogens the lone electron pair (marked in blue) is stable in its configuration and therefore the central sulfur atom now features four different substituents, making it a chiral center. In enzymatically produced SAM, only the biologically active *S*-isomer occurs. In synthetic SAM and its derivatives both isomers may be present, depending on the synthesis and purification methods. A mixture of a one-to-one ratio of both isomers is called a racemate or racemic mixture (49,58).

Exchange of the sulfur atom of SAM to selenium increased the electrophilicity of the adjacent side chain and thus the overall reactivity of the derivatives in methylation reactions. Simultaneously, selenium decreases the efficiency of at least three degradation pathways known from sulfur-SAM derivatives, as well as racemization (see Figure 3 and legend for a recapitulation of relevant chemical concepts) (50,51). These properties are even more pronounced in telluro derivatives. However, relative to sulfur, their electrophilicity is lower, leading to a lower reactivity in group transfer reactions. Therefore, their practical application is less interesting and consequently less investigated than for derivatives of sulfur or selenium (50,51).

The second approach was based on the observation that the group transfer rate can be increased by using β,γ-unsaturated SAM analogues (‘double-activated’ SAM analogues, Figure 2B). The transmethylation follows a bimolecular nucleophilic substitution (S_N_2) mechanism, implying the occurrence of an sp^2^ hybridized transition state on the α atom (52). Overlapping nonbonding orbitals from the central chalcogen are thought to stabilize this transition state *via* backbonding. Furthermore, the overlapping π-orbitals of an unsaturated β,γ bond present in synthetic SAM analogues are thought to stabilize this transition state.

The combination of both these activation mechanisms to enhance the reactivity of the α position towards nucleophilic attacks has coined the term ‘double activation’ (depicted in Figure 3A) (38,53). During the transfer reaction, the thus double activated alpha carbon in the side chain of SAM derivatives is attacked by a nucleophilic residue of the substrate molecule.

Utilizing this **m**ethyltransferase-directed **t**ransfer of **a**ctivated **g**roups (mTAG), group transfers of various reactive groups (54–56) as well as direct labelling with fluorophores (57) to DNA have been described, which are here only mentioned in passing. Conceptually, those are expected to be transferable to RNA substrates, although this has been put into practice only for a limited number, which are described in the following paragraph.

## RNA

### Synthesis of SAM analogues

Chemical synthesis of SAM analogues commonly starts from *S*-adenosyl-l-homocystein (SAH) or its respective analogues. The central chalcogen typically acts as a nucleophile in a simple nucleophilic substitution, usually of the S_N_2 type, with e.g. an alkylhalogenide as electrophile. A major drawback of this reaction is the racemic nature (Figure 3) of the resulting product, with only the *S* isomer being biologically active (Figure 3B). This may necessitate an additional challenging purification step, which lowers the overall yield (49,58).

Enzymatic synthesis of SAM derivatives circumvents the stereochemistry problem and thus presents a viable alternative, whose feasibility was demonstrated with various enzymes. The chlorinase SaIL and fluorinases FDAS are degrading SAM *in vivo*, but their reactivity can be inverted to synthesize a diverse set of SAM analogues using the corresponding methionine analogues as starting points. Bioengineered variants of these enzymes are able to accept even voluminous residues and the SAM preparation can directly be coupled to alkylation reactions using various MTases, like protein or small molecule MTases (59,60).

**Figure 4.**
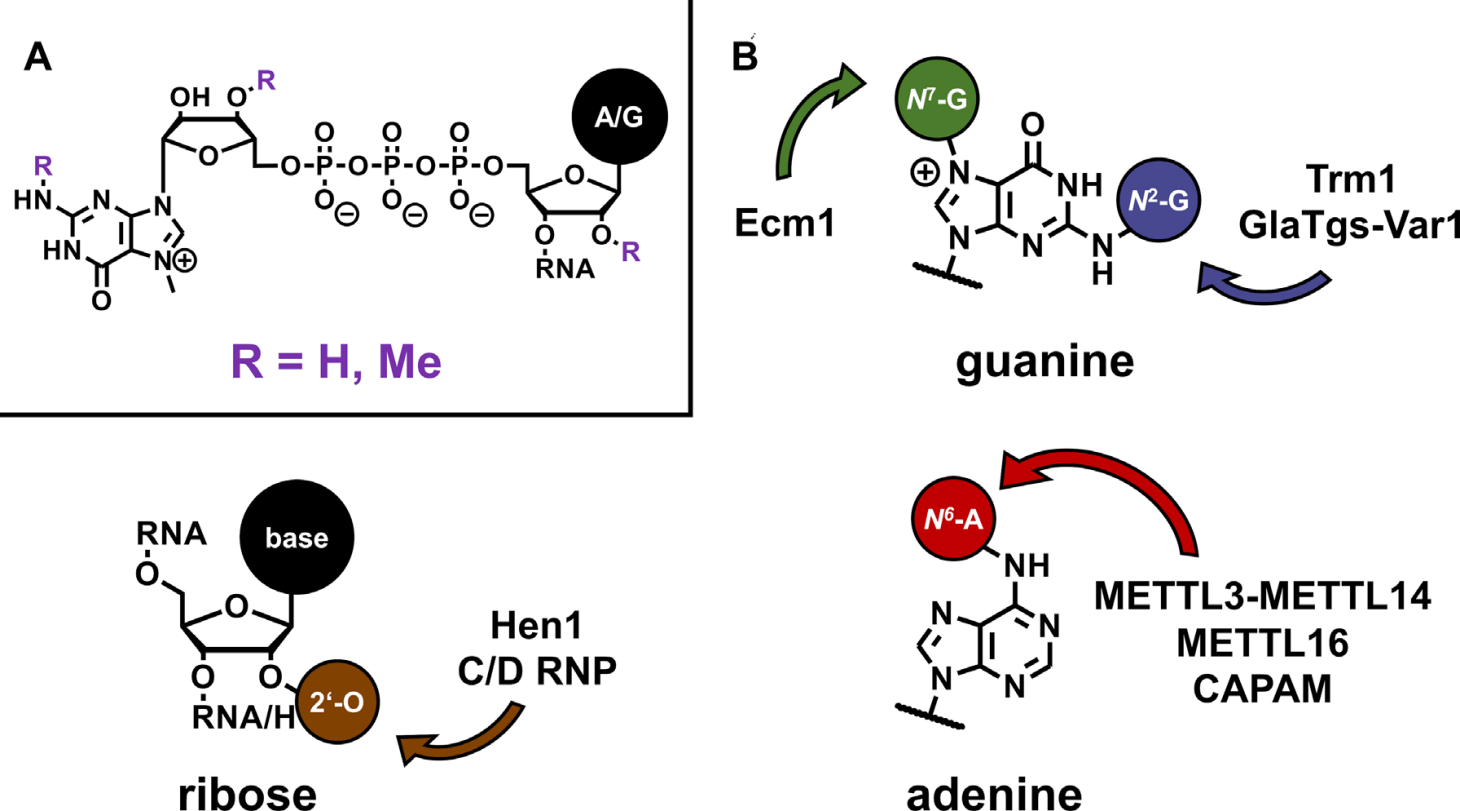
RNA structures targeted for functional group transfer. (**A**) General RNA cap structure. (**B**) Positions on RNA targeted with mTAG by various enzymes.

The human enzyme Methionine Adenosyltransferase (MAT) was engineered to act more promiscuously in the preparation of artificial SAM analogues. This enzyme family's biological role is to synthesize SAM from ATP and methionine. Through enzymatic cascade reactions, starting from the corresponding methionine analogues, various groups were not only transferred to small molecules, proteins and DNA, but also to RNA (61–64). Using an engineered MAT enzyme, suitable methionine analogues and the RNA MTases Ecm1 and GlaTgs2-Var1 RNA caps (general structure depicted in Figure 4A) were functionalized with various groups, such as azides, or terminal triple or double bonds.

SAM analogues featuring a central selenium atom were well accepted (64,65). Since, in contrast to SAM analogues, methionine and its analogues are cell permeable, RNA was directly labelled in cells using a seleno analogue of methionine bearing a propargylic group (65).

The MAT from *Cryptosporidium hominis* (ChMAT) was also used to synthesize SAM derivatives for subsequent MTase mediated labelling of minimal RNA-cap analogues, either in its native form (66) or as engineered variant for the transfer of bulky photocaging groups (PC-ChMAT) (63).

### Click chemistry

**Figure 5.**
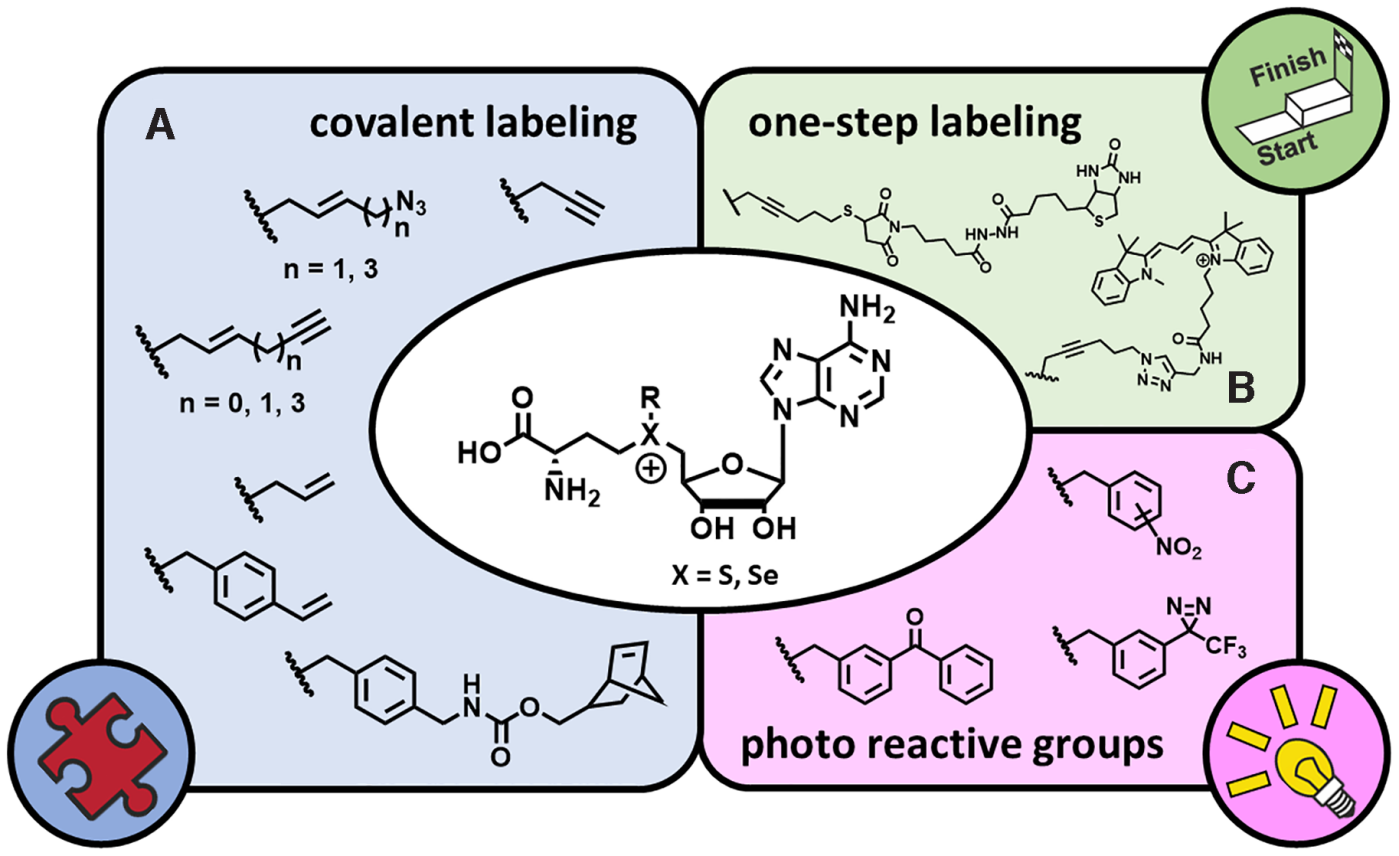
Typical residues transferred to RNA by mTag (**A**) for subsequent covalent labelling, (**B**) as photoreactive groups or (**C**) as one-step labelling.

A number of concepts from DNA labelling by SAM-analogues have been adapted for group transfer onto RNA using various RNA MTases, portrayed in Figure 4B. Among the variety of transferred groups, most were designed for subsequent labelling via ‘click’ chemistry (67–69) (Figure 5A). Within the field of click chemistry, the majority of reactions have by now been performed with RNA as a substrate, and consequently, most of the functional groups prominent in click chemistry have been included in substrates for transfer to RNA.

Together with the steadily increasing toolbox of enzymes used for RNA labelling, they allow site-specific-functionalization of various RNA species, including dual modified RNA. The latter was prepared using combinations of different enzymes and orthogonal click reactions (70).

**Figure 6.**
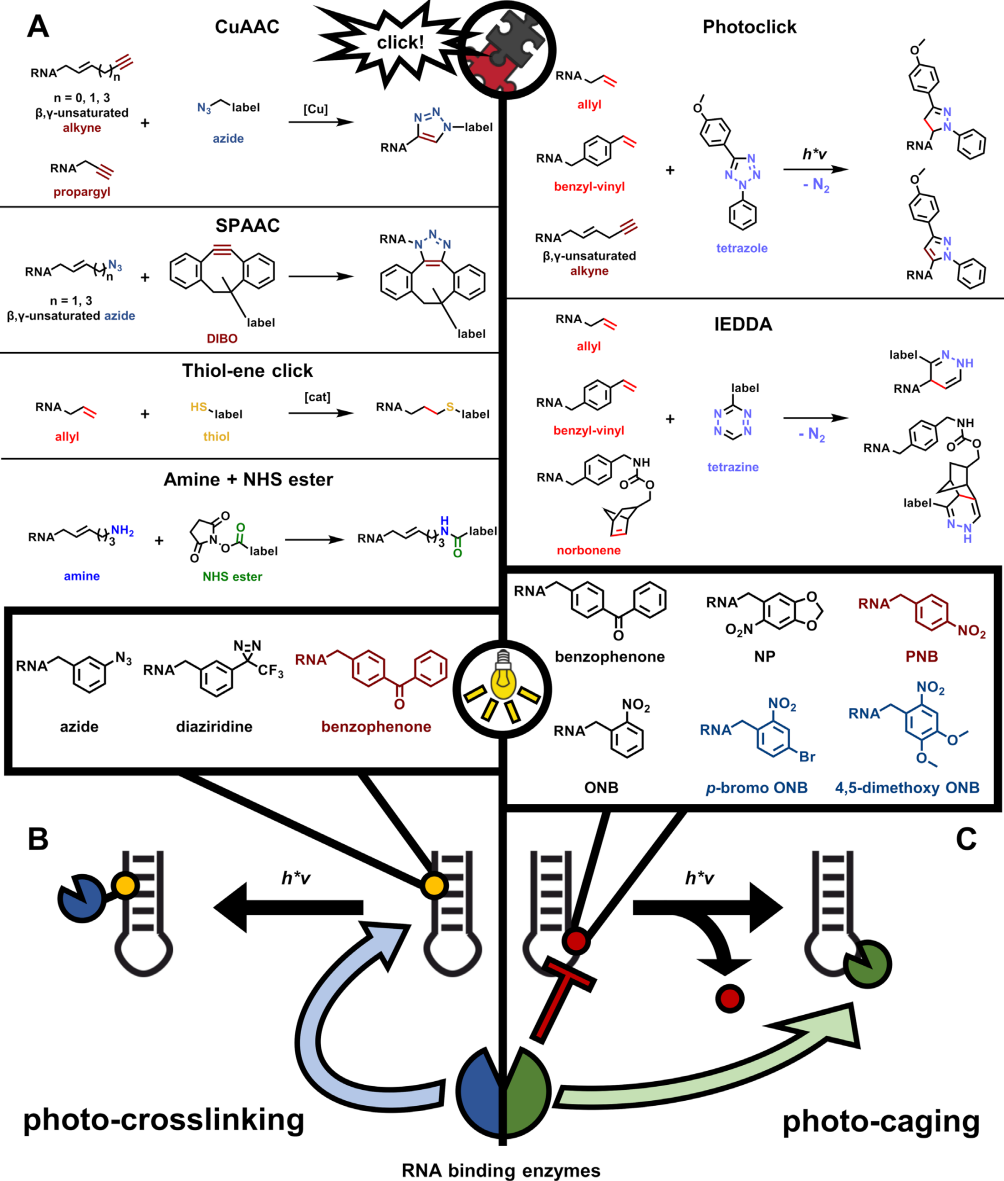
Overview of click, crosslink, and photo-caging. (**A**) Bioconjugation reactions used to attach mTAG modified RNA to chemical labels. Label moieties include fluorophores or biotin containing groups. CuAAC = copper catalyzed azide-alkyne 1,3-cycloaddition; SPAAC = strain-promoted azide-alkyne 1,3-cycloaddition; NHS = *N*-hydroxysuccinimide; IEDDA = inverse electron demand diels-alder. (**B**) Schematic representation of RNA photo-crosslinking together with molecular structures of photo-crosslinking groups transferred to RNA by mTAG. Molecules marked in red have been transferred to RNA but were not successful in photo-crosslinking. (**C**) Schematic representation of photo-caging with representative molecular structures of photo-caging groups transferred to RNA by mTAG. Molecules marked in red have been transferred to RNA but were not successful in photo-caging. Molecules marked in blue could have been transferred to RNA but their potential for photo-caging was only investigated with DNA.

The first group transfer using mTAG for RNA was described using eukaryotic tRNA-MTase Trm1. The SAM analogue used here contained an activating β,γ-double bond and a terminal alkyne. The latter moiety was thus transferred to the *N*^2^ of guanosine 26 on tRNA^Phe^ and subsequently labelled with an azide derivative of a fluorescent dye via the copper catalyzed azide-alkyne 1,3-cycloaddition (CuAAC, Figure 6A). The click adduct was confirmed by liquid chromatography–mass spectrometry (LC–MS) and the labelled tRNA was found suitable for application in single molecule spectroscopy (71). Numerous other group transfers utilizing SAM analogues with terminal alkynes variably linked to β,γ-double bonds have been described so far.

A variant of the trimethylguanosinsynthase from *Giardia lamblia* (GlaTgs2-Var1) with a V34A mutation (72,73), was reported to transfer these residues to the *N*^2^ position of m^7^G-caps (64,70,72,74,75). The *N*^7^ position of guanosines in caps was found accessible for labelling using the Ecm1 enzyme (70,76). Furthermore, *N*^6^ positions of adenine bases were modified at least in traces with terminal alkynes containing β,γ-double bonds using the METTLL3-METTL14 or METTL16 enzymes (77). The HEN1 enzyme from *Arabidopsis thaliana* was employed for transfer to the 2′-OH group on the 3′ end of small, double-stranded RNAs or RNA–DNA duplexes (78,79).

Subsequent CuAAC reactions transferred fluorescent dyes (64,72,74) or biotin groups (64) to yield functionalized RNA. Numerous alternatives to the ‘classical’ CuAAC click reaction have meanwhile been developed to avoid the use of copper ions. Among these alternatives, a photoclick reaction was implemented to attach fluorescent dyes to alkyne modified RNA cap analogues (75) (Figure 6A).

The various RNA MTases that would catalyze transfer of propargylic residues included GlaTgs2-Var1 (64,70), as well as Ecm1 (70), METTL3-METTL14 (65,77), and METTL16 (77). As described above, resulting terminal alkynes could be modified using the CuAAC click reaction. Thus, not only fluorescent dyes (64,70,80) and biotin (64,65) could be attached to the RNA, but also sterically demanding chemical groups (65). These bulky residues block the reverse transcription at the RNA label site, which could be utilized for the characterization of enzyme specificity and detection of unknown modification sites.

The fact that selenium analogues of SAM can act as suitable and even better substrates for RNA MTases was shown by using an enzymatic cascade with a MAT-Var and GlaTgs2-Var1 on the RNA cap analogue m^7^GpppA. For a terminal alkyne, containing a β,γ-double bond, at least a moderate increase of incorporation was observed. When exchanging the sulfur atom to selenium in the SAM analogue bearing a propargylic residue, the incorporation efficiency rose strongly from the low two-digit percentage range to near quantitative incorporation (64). These findings are in line with data from a protein MTase, which demonstrates that propargylic residues are better transferred when using the corresponding selenium derivative instead of the sulfur one (81).

Aside from terminal alkynes, residues bearing azides were transferred to RNA in substantial yield using the MTases GlaTgs2-Var1 (64,70,74,76,82), Ecm1 (70,83) and HEN1 (79,84), while the adenine-*N*^6^ modifying enzymes METTL3-METTL14 and METTL16 on the other hand were only able to transfer traces of azides so far (65,77). Azide labelled RNA has been tagged using the CuAAC as described above, with alkyne bearing fluorescent dyes (79,84). As a copper-ion-free alternative, the strain-promoted azide-alkyne 1,3-cycloadditions (SPAAC, Figure 6A) used the strained alkyne residue dibenzocyclooctyne (DIBO) for functionalization of RNA with fluorescent dyes (64,70,74,76,82,83) or biotin (70).

Within the selection of click reactions the attachment of double bonds to RNAs is also of interest, since these can be functionalized using thiol-ene, inverse electron demand diels-alder (IEDDA), and specific photoclick reactions (Figure 6A). For this purpose, allyl groups were conjugated to RNAs by the enzymes GlaTgs2-Var1 (64,70,72,73,75,85), Ecm1 (70,76) and METTL3-METTL14 (65). These moieties were further modified either with biotin in a thiol-ene reaction (72), or with fluorescent dyes using IEDDA (85) or photoclick reactions (59,69).

Another double bond bearing group transferred to RNA is the benzyl-vinyl group, which was introduced to RNA with GlaTgs2-Var1 (70,74,85), Ecm1 (70,76) and METTL3-METTL14 in satisfying yields (77). Similar to allyl groups, fluorophores were attached by IEDDA (70,74,76,85) and photoclick reactions (76,85).

Interestingly, even bulky residues such as norbornene derivatives with benzylic linkers have been successfully transferred from corresponding SAM analogues to RNA caps with the Ecm1 enzyme. The norbornene double bond was exploited for the attachment of biotin or fluorescence labels using the IEDDA reaction (83).

Beyond classical click reactions in the proper sense, reactions of amines with *N*-hydroxysuccinimide (NHS, Figure 6A) esters are popular bioconjugations, which are frequently exploited for protein and nucleic acid functionalization (86). This reaction was also used to modify RNA with fluorescent dyes or biotin, after transferring amino groups with *A. thaliana* and *D. melanogaster* HEN1 (78,79,84).

A highly interesting development concerned the substrate RNA specificity of enzymes employed in transfer reactions. Most of the used MTases typically display high target selectivity, thus limiting the targetable range of RNA species, residues, and functional groups within the latter. Not only is there no free choice of positional labelling, but in potential *in vivo* applications, an enzyme must also compete with native MTases for RNA substrates, which could limit the application. This makes the C/D small ribonucleoprotein RNA 2′-*O*-methyltransferase (C/D RNP) particular interesting, since its substrate specificity is programmable via a guide RNA and can thus be targeted to variable sites on RNA where it modifies 2′-*O* residues. This way, a pre-mRNA and tRNA^Leu^ could be modified with a propargylic residue at various sites, each addressed by suitable guide RNAs and the corresponding seleno-derivative of SAM (80).

In summary of the above, various divergent functional groups were shown to be transferable from their respective SAM analogues to different RNAs using the mTAG approach. Subsequent derivatization with all major types of click chemistries enabled RNA functionalization not only *in vitro*, but also *in vivo*. For the latter, the labelling was based on the use of methionine analogues and MAT enzymes. While the GlaTgs2-Var1, Ecm1 and HEN1 enzymes have been investigated quite thoroughly, the scope of MTases exploited for mTAG remains rather limited. This might hinder the use of mTAG if no suitable enzyme for a given substrate is available. Thus, detection and development of novel RNA modifying enzymes for mTAG remains a desirable goal.

### One-step transfer concepts

In all of the above mentioned cases the mTAG modified RNA must undergo a secondary click reaction step to attach the desired label, which exposes the RNA to potentially damaging chemical conditions. In a more gentle approach, fluorescent dyes or biotin were attached to the side chains of SAM-derivatives, before the enzymatic transfer to RNA (Figure 5). These voluminous side chains were then grafted onto the RNA by the *A. thaliana* and *D. melanogaster* HEN1 enzymes (78,79,84).

### Photo-crosslinking and photo-caging

A number of SAM derivatives have been designed for transfer reactions of photoactivatable groups, which were typically used for the characterization of RNA-protein complexes (Figure 5). One important application is photo-crosslinking which can be conducted with native RNA, thereby causing crosslinks to its interacting proteins upon irradiation with UV-light. The introduction of more reactive photoactivatable groups can overcome a number of drawbacks which are encountered e.g. in techniques using low wavelength UV light of e.g. at 254 nm, such as UV crosslinking with subsequent Immunoprecipitation (CLIP) (87). These drawbacks include an overall low yield, photo-induced RNA strand breaks, inter- or intramolecular crosslinking of the RNA, and varying reactivities of different nucleotides and amino acids (88,89).

Well established photoreactive groups include aryl azides, trifluoromethyl phenyl diaziridines, and benzophenones (90), and all of these have been employed in SAM analogues and corresponding transfer reactions to RNA caps using Ecm1 (Figure 6B). Wavelengths used were still in the UV range, e.g. 312 nm (azides) or 365 nm (diaziridines and benzophenones), but out of the absorption range of RNA. While azide- and diaziridine-containing RNA-caps could be crosslinked successfully to the eukaryotic translation initiation factor eIF4, benzophenone-modified RNA was not able to crosslink, presumably due to the bulkiness of the group (91,92).

The same bulkiness of the benzophenone group was instead exploited for photo-caging in the same context: in photo-caging, photo-labile groups block interactions or activity, which are restored upon removal of the caging group by irradiation (93,94). In the case at hand, the affinity of Ecm1-modified capped RNA analogues to the RNA-interacting enzymes eIF4E and DcpS was restored upon removal of the benzophenone by irradiation at 365 nm (95) (Figure 6C). Light dependent uncaging of the benzophenone group was also possible when internal RNA positions were chemically labelled at the *N*^7^ position of guanosine.

Other prominent photo-caging groups like ortho-nitrobenzyl (ONB) and para-nitrobenzyl (PNB), were successfully transferred from SAM derivatives to RNA cap analogues by Ecm1 but could not be removed upon irradiation (95). ONB, as well as a bromo and dimethoxy derivative could also be attached to an RNA cap analogue in an enzymatic cascade starting from corresponding methionine analogues and the above mentioned PC-ChMAT in combination with Ecm1 (63). Using a mutated MjMAT from *Methanocaldococcus jannaschii* and Ecm1 ONB, *p*-bromo ONB and 4,5-dimethoxy ONB residues could be transferred to RNA caps with higher yields compared to the reaction with PC-ChMAT and Ecm1 (96).

Internal RNA modification with photocaging groups was achieved by using METTL3-METTL14, which was able to transfer ONB and a 6-nitropiperonyl group (NP) to model mRNAs. Contrary to the findings with Ecm1 modified RNA caps, both groups could be removed successfully after irradiation with wavelengths of 365 or 405 nm (ONB) and 420 nm (NP). Furthermore, the ONB modification blocked reverse transcriptase activity on the labelled RNA, which was restored after light induced uncaging (77).

As for chemical reactive groups, various photoreactive groups have been transferred to RNAs, the extent of used enzymes however, is very limited. Since the requirement of spacious conjugated π electron systems for photoreactivity naturally increases the size of the residues, the requirements for the MTases are more demanding, making this limitation understandable. Nevertheless, a broader selection of enzymes might be wish worthy since the high utility of photoreactive groups in RNA research.

### Advanced applications

Beyond proof-of-concept studies for transfer of the various functional groups, the mTAG approach has been applied to a number of biological questions. One such application addressed the methylation specificity of the METTL3-METTL14. Using recombinant enzymes, allyl side chains of a corresponding SAM derivative were transferred to mRNA. Subsequent treatment with iodine led to *N*^1^-*N*^6^-cyclization of the modified adenine base but was inert with respect to unmodified adenosines. The resulting bulky tricyclic RNA modification caused misincorporation during reverse transcription, which was then identified by high-throughput sequencing. This technique thus revealed the site-specificity of alkylation by the METTL3–METTL14, implying similar specificity in the biogenesis of m^6^A (97).

In a similar objective, propargyl groups were transferred from seleno derivatives to RNA using the METTL3–METTL14 complex. After conjugation of the propargylic group to biotin with the CuAAC, the RNA was pulled down by streptavidin coated beads. The following reverse transcription, followed by Illumina sequencing of the cDNA, showed high stop ratios at the positions which were interpreted as modification sites along the same lines as above. Since methionine analogues are cell permeable and are metabolized to the corresponding SAM derivatives, this method can be used in living cells (65).

Cap modifications are currently of increased interest because they are known to modulate both translation efficiency and immunogenicity of mRNAs, including in particular mRNA vaccines. The ability of Ecm1 and GlaTgs2-Var1 to modify mRNA caps was exploited to tune the translation efficiency of synthetic mRNA by incorporating unnatural cap modifications. In most cases, this led to an abolishment of translation, however, caps modified with *N*^2^-allyl and *N*^7^-benzyl residues sustained moderate protein synthesis (76).

Another cap modifying enzyme applied for mTAG is CAPAM, which further modifies 2′-*O* methylated adenosines next to the m^7^G-cap, leading to a m^6^A_m_ modification. While this methylation leads to a strong decrease in translation, an incorporated propargyl residue from the corresponding seleno SAM derivative on this position only moderately impeded protein biosynthesis. Furthermore, the *N*^6^ propargyl modification significantly increased the innate immune response relative to unmodified adenosine, making it an interesting tool for the tuning of mRNA vaccines (98).

Via their multiple modification sites, RNA caps offer the opportunity for multiple labelling. This was exploited using Ecm1 and GlaTgs2-Var1, to attach two functional groups on the same cap in several permutations: either double labelling with the same fluorescent dye, or with a förster resonance energy transfer (FRET) pair, or with biotin and a fluorescent dye (70). Since mRNA can be modified by METTL3–METTL14 and METTL16 using mTAG, a similar approach could, in principle, be applied here as well (77).

One obvious problem in using mTAG for RNA labelling, especially *in vivo*, is the potentially low selectivity of SAM analogues in the presence of multiple MTases. To tackle this, ATP analogues with a derivatized adenine bases and 2′-*O* positions were prepared. In enzymatic cascade reactions using MAT and various MTases, these were first transformed to the corresponding SAM analogues, which then transferred their residues onto the RNA. Remarkably, partial selectivity towards certain enzymes was achieved with specific residues (66).

### Highlights of RNA MTase chemical biology

The above mentioned examples clearly demonstrate the benefit of RNA MTase mediated labelling. For the use of mTAG in RNA research, cap modifying enzymes have been the most utilized. Even in its native form, the Ecm1 enzyme has proven highly promiscuous and was shown to transfer a remarkable variety of chemical and photoreactive groups to RNA caps in high yields. Just as remarkable, the HEN1 enzymes of *A. thaliana* and *D. melanogaster* were not only able to attach numerous reactive groups to RNA from the corresponding SAM analoga but can also perform single-step transfer of voluminous side groups bearing fluorescent dyes or biotin.

In our perception, the field is poised to move to a new level of usefulness to the wider community. Current objectives revolved around the enzyme's characteristics with respect to cofactor promiscuity versus specificity on one hand, and the targeting of specific RNA moieties or sequences on the other hand. While cap modifying enzymes are best characterized for the former aspect, their target residue in RNA is essentially invariant. Here, MTase activities featuring guide RNAs offer a promising opportunity for further development. As an example, MTase activity on C/D RNPs can be programmed with a guide RNA and accordingly could be made to modify various sites in different RNAs almost at will (80). Remarkably, not only C/D RNP activity can be directed by guide RNAs, but also the activity of RNA acetylases like Kre33 or Nat10, acetylating cytosins and implementing ac^4^C modifications, which might expand the scope for site specific RNA labelling concepts in the future (99,100).

## INHIBITION OF RNA MTASES BY SMALL MOLECULES

A survey of published literature reveals that the number of known inhibitors of MTases in general (101–103) and RNA MTases in particular is relatively limited (104). As already stated, this is in contrast to other transferase enzyme families, such as kinases (3–5). Although not quite as abundant as ATP, SAM is still a widespread cofactor, and inhibition of methyltransfer activity offers numerous perspectives for therapeutic intervention. DNA MTases have been targeted for small molecule drug development mostly in the perspective of developing antibiotics or anti-cancer drugs (105,106). This holds true for RNA methyltransferases (107,108), which, in addition have been identified as an attractive target for antiviral drugs (109).

### Assay development

On a general level, development of small molecule inhibitors for methyltransferases may follow one of several well-established approaches. Among other things, the choice of approach will depend on the availability of a precise and accurate assay. In a conventional methyl transfer reaction, one may e.g. target the formation of the by-product SAH (110,111). Furthermore, depending on the type of methyltransferase, quantification of the methylated target molecule is an option (110,112–114). While the former approach lends itself to the development of generic assays, which may be applied to essentially any methyltransferase, the latter one must be specifically adapted to each individual enzyme.

Generic detection schemes for methylated products frequently rely on mass spectrometry, but occasionally, typically in well studied systems, specific substrates have been developed that the use of detection methods other than mass spectrometry. One such is the synthesis of a fluorescent cap structure analogue as an accepted substrate for *N*^7^-G mRNA cap MTases, which changes its fluorescence properties (114).

Quantification of the generic co-substrate SAM and its metabolite SAH commonly relies on separation by high-performance liquid chromatography (HPLC). After the methylation reaction, macromolecules like enzymes and RNA are typically precipitated, followed by chromatographic analysis of the supernatant. Chromatographic quantification methods for SAM and SAH have been described in conjunction with either UV (115), fluorescence (116–118), mass spectrometry (119), or electrochemical detection (120). However, the time-consuming nature of such an end-point analysis limits its use to low or medium sample throughput. While such methods can be refined to perform with appreciable accuracy, reproducibility, and sensitivity, the required instrumentation imposes an intrinsic limit to throughput numbers.

For the development of techniques with higher throughput in combination with HPLC, e.g. in a 96-well plate format or higher, a staggered parallel scheme was developed. In this approach, multiple HPLC systems are connected to one mass-spectrometer and are run with a time delay to allow the MS system to switch to incoming analytes, thus minimizing the unused time of the detector (121,122).

In the RapidFire approach the HPLC column is replaced by an automated solid-phase-extraction. This lowers the throughput time for the purification drastically before analytes are quantified with a mass-spectrometer (123,124). RapidFire-MS/MS was indeed used for larger screenings to identify novel RNA MTase inhibitors (125,126).

Plate formats are also frequently applied for spectrophotometric methods, thus methods suitable for plate reader equipment are desirable, that typically rely on either absorption (127), luminescence (128) or fluorescence (129–131). Given that SAM and SAH consist of generic metabolic building blocks with commonplace spectroscopic properties, their specific quantification by spectroscopy constitutes a challenge. Hence, it is common to enzymatically convert the produced SAH into metabolites for which specific detection schemes have already been established.

Such pathways have been established for both constituents of SAH, namely homocysteine and adenine, either of which can be converted into better chromophores to generate a downstream spectrometric signal. Enzymatic lysis of SAH catalyzed by adenosylhomocysteinase (AHCY) or a combination of AdoHcy nucleosidase (mtnN) and *S*-ribosylhomocysteine lyase (LuxS) releases homocysteine which can be spectrometrically quantified after coupling to reactive stains like ThioGlo1, DTNB, or monobromobimane (132,133).

On the other hand, adenine released by cleavage through mtnN, can further be converted into hypoxanthine via adenine deaminase, and then spectrometrically quantified (134). Alternatively, ATP enzymatically generated out of adenine was fed into a bioluminescence assay, where a combination of a total of four enzymes were ultimately coupled to a luciferin/luciferase reaction (135,136).

The advantage of high-throughput numbers offered by concatenated enzymatic conversion methods is partially offset by potential side reactions and interfering substances in each enzymatic conversion. Hence, such assays frequently come into play when an MTase system is already well understood, and the time required for their optimization correlates with an expected return from high-throughput screening (HTS).

In early assay development and low-throughput screening (LTS), the arguably most direct and robust way to detect a methylation reaction consists in tracing isotope-labelled methylgroups during their transfer from SAM onto the substrate. A widespread method for RNA MTases is based on radioactive labelled ^3^H-SAM. After separation from the tritiated cosubstrate, methylated product can be directly quantified via scintillation counting. For example, methylated RNA can be separated from ^3^H-SAM by precipitation with trichloroacetic acid (TCA) on paper (137). Small fragments or nucleotides, including digested substrate RNA, can be quantified by imaging of the radioactive labelled reaction product after separation by thin-layer chromatography (138–140).

Scintillation proximity assays (SPA) represent an approach to improve throughput numbers when working with radioactivity. Using a similar detection principle, these can be carried out in 96 or higher well plate formats. Methylated, tritium-containing RNA is being separated from remaining labelled SAM by binding to scintillator beads. Binding can be mediated by unspecific electrostatic interactions or by exploiting specific affinity pairs such as biotin/streptavidin when using biotinylated substrate RNA. Radioactive decay from tritium in proximity to the beads then causes excitation of the scintillator and a subsequent emission that allows quantification of bound tritiated RNA (141–143).

### Provenance of RNA MTase Inhibitors

**Figure 7.**
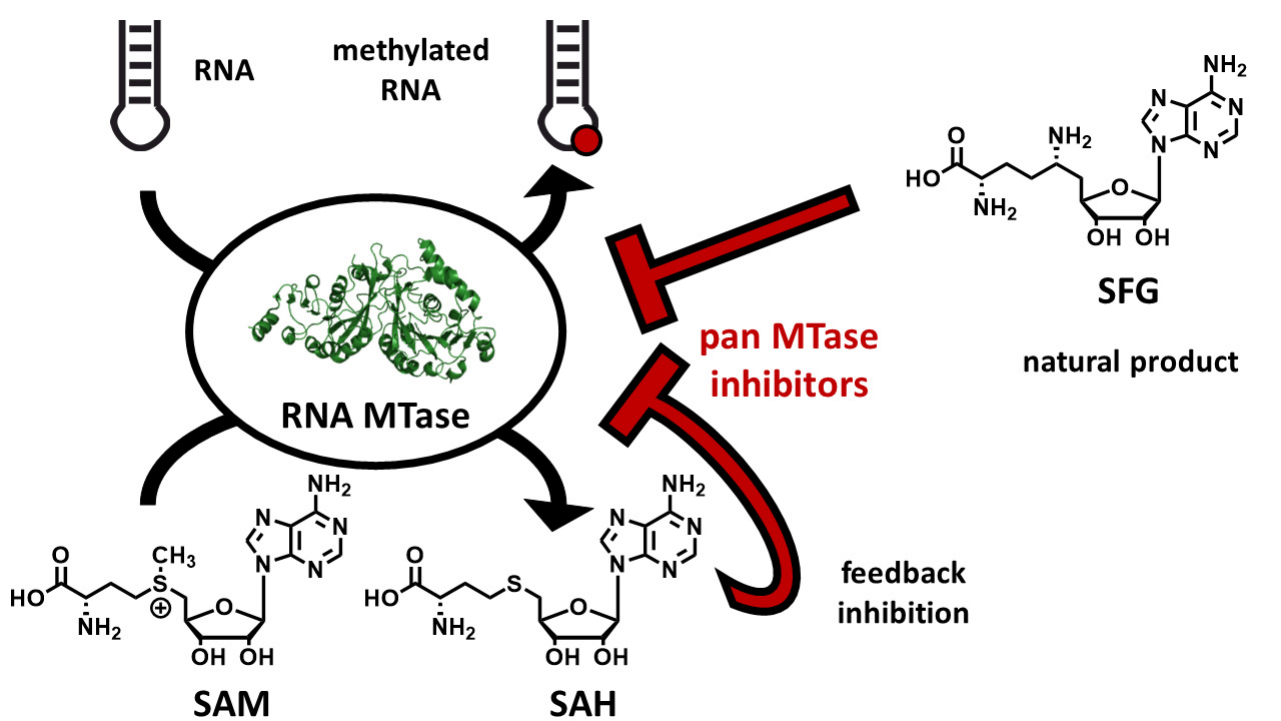
Natural product pan-MTase-inhibitors. The byproduct of the methylation reaction, SAH, acts as a feedback inhibitor. SFG is a natural product extracted from *Streptomyces griseolus*. Both molecules are well known pan-MTase-inhibitors. Protein structure created with PyMOL (Schrödinger LLC (2010) The PyMOL Molecular Graphics System, Version 1.3.), PDB ID used: 5IL2 (281).

Many of the currently known RNA MTase inhibitors have been developed as substrate analogues, either by uninformed library screening, or by screening of focussed libraries, compiled with the help of computer-aided docking studies. Prior to the advent of X-ray structures, which might have allowed the design of non-substrate analogues as inhibitors, numerous derivatives of SAM and of SAH, itself an efficient inhibitor of many enzymes, were tested (Figure 7). Such substrate analogues would be expected to effect inhibition by competing with SAM for the respective binding pocket.

In addition to SAH (144), sinefungin (SFG) was found to inhibit most MTases investigated (145). As a SAM analogue that was first isolated from *Streptomyces griseolus*, SFG features a central nitrogen *en lieu* of the typical trivalent sulfur atom and is therefore more stable against hydrolysis (Figure 7). Both compounds are often used as inhibition controls in assay development and frequently serve as starting points for inhibitor design (111). This approach was also utilized for the design of inhibitors for various RNA MTases and thus will be discussed in detail below.

Recent developments of MTase inhibitors in the RNA field are based on some general findings dating back about half a century ago. Back then, correlations between RNA methylation and the spread of cancer, as well as the infectiousness of viruses had already been established (146,147). Consequently, attempts to modulate the enzymatic activity of RNA MTases are nearly as old (148).

Prior to the advent of technologies for recombinant protein expression, several closely related derivatives of the above-mentioned pan MTase inhibitors SFG and SAH were typically tested against the MTase activities of crude enzymatic extracts (149–157). These inhibitors typically show moderate potency against their respective targets at best, but first cases of selectivity towards specific enzymes were already observed (158,159). Furthermore, early cases of structure activity relationships were established against specific enzymatic extracts with RNA MTase activity (160–162).

Yet another important concept that was already pursued was the use of bivalent inhibitors that would address the separate binding sites for SAM and RNA with a single small molecule (163). This idea was taken up recently for the design of inhibitors e.g. against *severe acute respiratory syndrome coronavirus 2* (SARS-CoV-2) nsp14 or RlmJ (164–166).

### Screening approaches

For the identification of novel ligands or inhibitors for a given target, different strategies have been developed, which differ significantly with respect to throughput numbers. In high-throughput screening (HTS) campaigns up to millions of compounds can be tested to find new hits, provided a robust and sensitive assay format is available (167). A different approach to identify novel inhibitors is fragment-based drug discovery (FBDD). Here, molecules with limited molecular weight (< 300 Da), which are considered fragments of a to-be-developed inhibitor compound, are screened by sensitive biophysical techniques, typically followed by structure determination of the target-hit complexes, e.g. X-ray crystallography (168). Fragment hits may be acceptable even with low binding affinities in the high micromolar to low millimolar range, provided they show high ligand efficiency, meaning that nearly every atom of a fragment contributes productively to the binding event (169).

In a next step, such fragments are then expanded based on the co-structure with their respective target. In this ‘fragment growing’ step, potential additional interactions in proximity to the fragment binding site are explored by addition of matching functional groups to the fragment. Provided the new functional groups do indeed improve binding parameters, this step can be repeated several times, leading to bigger compounds which usually have high affinities coupled with high ligand efficiencies.

Alternatively, multiple fragments binding to distinct sites can be linked in a search for synergy akin to bidentate ligands. Of course, the growth process outlined for fragments can be applied to the resulting multi-fragment compounds as well. Advantages relative to HTS include smaller libraries and better understanding of the chemical, physical and biological properties of compounds, as they emerge from the rational fragment growing process (168–170).

As a viable supplementary approach, different flavours of virtual screening (VS) approaches aim to select promising molecules *in silico* prior to experimental testing (171). Libraries used in VS approaches may contain 10^15^ and more molecules (172,173), which do not necessarily have to be physically available at the onset. Rather, their prospective make-on-demand synthesis needs to be plausible in case they emerge as promising from the VS. VS can further be divided into structure-based drug design (SBDD) or ligand-based drug design (LBDD). SBDD involves virtual molecular docking, thereby taking advantage of the knowledge of the target's three-dimensional structure. In contrast optimization in LBDD is based on the structures of known ligands (174).

As the success rates of both HTS and VS rely on the quality of the screened molecular libraries, general rules like drug-likeness, lead-likeness, or removal of unwanted (reactive) groups and awareness for pan-assay interference compounds (PAINS) are regularly applied in library design to avoid false-positive hits (175–179).

### Bacterial MTase inhibitors

The treatment of bacterial infections with multiple antibiotic resistances is nowadays a significant and growing challenge. New strategies involve both, overcoming resistance mechanisms, and developing antibiotics against new targets. Both strategies are also being pursued with prokaryotic RNA methyltransferases as targets, which are displayed in Table 2.

**Table 2.** List of small molecule inhibitors for prokaryotic RNA MTases. The ‘design strategy’ column describes the basic method for inhibitor design, while ‘evaluation method’ roughly lists the experiments conducted in the associated references. CADD = computer aided drug design, i.s. = *in silico*; i.vt. = *in vitro*; i.c. = *in cellulo*; i.vv. = *in vivo*

Enzyme/ Modification/ RNA speceis	Organism	Design strategy	Evaluation method	References
ErmC/ m^6^A/ rRNA	*E. coli*	CADD	i.s., i.vt.	(184)
		CADD	i.s., i.vt.	(183)
	??	CADD	i.s., i.vt.	(182)
	*Staphylococcus aureus/pyogenes*	HTS	i.vt., i.vv.	(185)
ErmAM/ m^6^A/ rRNA	??	NMR-based screening	i.vt.	(186)
TrmD/ m^1^G_37_/ tRNA	*E.coli*	SAH analogue	i.vt.	(187)
		HTS	i.vt.	(142)
	*Pseudomonas aeruginosa*	HTS	i.vt.	(108)
		SAR, non SAH-like	i.vt.	(188)
	*Mycobacterium tuberculosis*	SAR, non SAH-like	i.vt.	(188)
	*Mycobacterium tub. + abscessus + leprae*	FBDD	i.vt., i.c.	(189)
	*Haemophilus influenzae*	HTS	i.vt.	(142)
	*S. aureus*	HTS	i.vt.	(142)
		SAR, non SAH-like	i.vt.	(188)
	*Mycobacterium abscessus*	FBDD	i.vt.	(190)
crude extract/ m^5^U/ tRNA	*E. coli*	SAH analogue	i.vt.	(160)
RlmJ/ m^6^A/ rRNA	*E. coli*	SAH analogue	i.vt.	(166)
crude extract/ unknwon modification/ tRNA		Purine & adenosine analogs	i.vt.	(193)
crude extract/ m^1^A, m^6^A, m^6^_2_A, m^7^G/ tRNA, rRNA	*Streptomyces*	SAH analogue	i.vt.	(162)
Not specified		SFG, Azacytidin	i.c.	(298)
Dimethyladenosine transferase/ m^6^A/ rRNA	*Chlamydia pneumoniae*	Similarity based VS	i.s., i.c.	(191)
rsmD like rRNA-MTase/ m^2^G/ rRNA	*Wolbachia*	CADD	i.s., i.vv.	(192)

**Figure 8.**
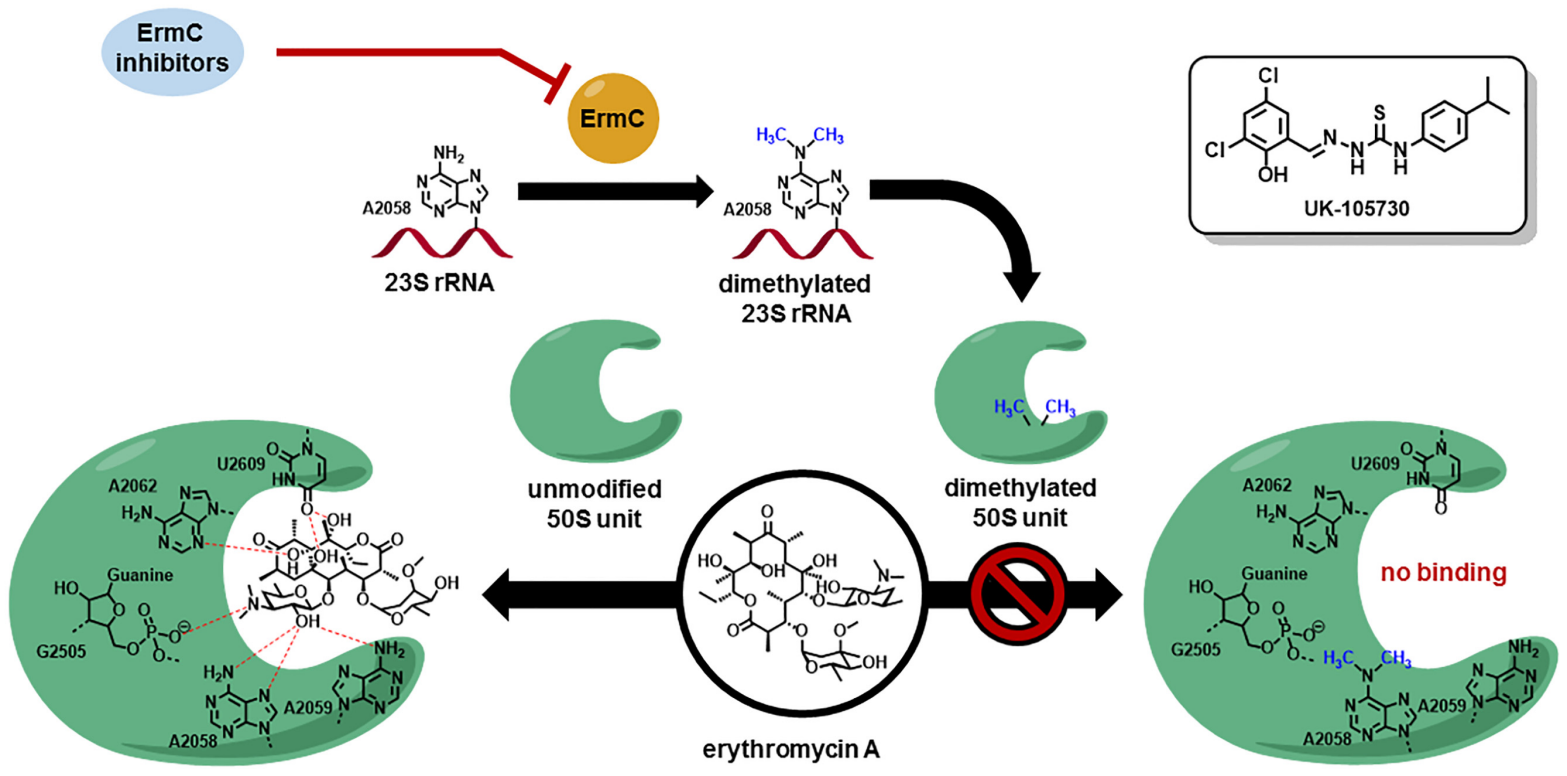
Function of ErmC and ErmC inhibitors. ErmC dimethylates A2058 of 23S rRNA incorporated into the 50S unit, thereby preventing the binding of erythromycin A. Potential Inhibitors of ErmC have potential in a combination therapy with macrolides by enabling the binding of the antibiotic. An inhibitor with affinity in the sub-micromolar range (UK-105730, IC_50_ = 0.45 μM) is depicted in the top right corner. Figure adapted from Steinhilber *et al.* (282).

An interesting target with respect to overcoming resistances is the erythromycin resistance methyltransferase (Erm) family, as their enzymes modify rRNA at binding sites for antibiotics such as macrolides, lincosamides and streptogramin, thus simultaneously causing resistance to multiple antibiotics (180) (Figure 8). Therefore, they represent promising targets for the treatment of bacterial infections by a combination therapy of Erm inhibitors and conventional antibiotics e.g. macrolides.

The ErmC enzyme is the best characterized member of this family (181). Utilizing different methods of virtual (182–184) and high-throughput (185) screenings, inhibitors of ErmC with moderate affinity (e.g. UK-105730 (Figure 8), half maximal inhibitory concentration (IC_50_ = 0.45 μM) could be identified. Inhibition showed the sensitization of resistant bacteria to azithromycin (185). These findings sound promising, however, the recent interest in such inhibitors is low and there are no signs for a medicinal use so far.

Another enzyme of the Erm family is ErmAM, for which inhibitors were identified by an nuclear magnetic resonance (NMR) screening approach. Optimisation of hits by parallel synthesis led to inhibitors in the low micromolar range that bind competitively in the SAM pocket (186).

Next to ErmC, which is an almost traditional target, TrmD, which modifies tRNA at *N*^1^ of G37, has been extensively studied in the more recent past. Various SAH-like as well as non-SAH-like inhibitors were tested against TrmD from different prokaryotes including *E. coli* (142,187), *P. aeruginosa* (108,188), *M. tuberculosis* (188,189), *H. influenzae* (142), *S. aureus* (142,188), and *M. abscessus* (190). Either SAR studies were conducted (187,188), or, in order to determine new structures, different approaches have been followed such as HTS (108,142) and FBDD (189,190).

Further targets for which inhibitors were developed include RlmJ in *E. coli* (166), a dimethyladenosine transferase in *C. pneumoniae* (191) and the rsmD like rRNA-MTase in *Wolbachia* (192). In early studies, the effect of inhibitors on the methylation of RNA was characterized in *E. coli* (160,193) and several strains of *Streptomyces* (162), however, specific targets were not determined.

### Eukaryotic single cell organisms as targets

Over decades, the development of RNA MTase inhibitors against yeast or single cell fungi has seen very low activity (Table 3). Sinefungin, whose name implies antifungal activity, has been tested on targets in fungi such as Ecm1 in *E. cuniculi* (114), and CCM1 in *C. albicans* (194). Additionally, it has been tested on ABD1 in yeast (*S. cerevisiae*) (194), and on Cmt1 in *L. infantum* (195).

**Table 3.** List of small molecule inhibitors for eukaryotic RNA MTases. The ‘design strategy’ column describes the basic method for inhibitor design, while ‘evaluation method’ roughly lists the experiments conducted in the associated references. CADD = computer aided drug design, i.s. = *in silico*; i.vt. = *in vitro*; i.c. = *in cellulo*; i.vv. = *in vivo*

Type	Organism	Enzyme/Modification/RNA species	Design strategy	Evaluation method	References
**Eukaryotes (single cell)**	Fungi, *Encephalitozoon cuniculi*	Ecm1/ m^7^G/ mRNA cap	SAH analogue	i.vt., i.c.	(197,198)
			Nucleotide analogue	i.vt.	(114)
	Fungi *C. albicans*	CCM1/ m^7^G/ mRNA cap	Natural products	i.c.	(194)
	Yeast *S. cerevisiae*	ABD1 (*S.c*.)/ m^7^G/ mRNA cap	Natural products	i.c.	(194)
	*Leishmania infantum*	Cmt1/ m^7^G/ mRNA cap	SAH analogue	i.vt.	(195)
	*Trypanosoma brucei*	Cmt1/ m^7^G/ mRNA cap	SAH analogue	i.vt.	(196)
**Eukaryotes (multiple cell)**	Human, *H. sapiens*	METTL3-METTL14/ m^6^A/ mRNA internal	CADD	i.s., i.vt., i.c.	(223)
			SAH analogue	i.vt.	(166)
				i.s., i.vt.	(221)
				i.s., i.vt., i.c.	(222)
				i.vt., i.c.	(299)
			Non SAH analogue	i.s., i.vt., i.c., i.vv.	(126)
				i.vt., i.c.	(216,224,300)

Next to sinefungin, only few inhibitor classes were investigated as potential inhibitors of eukaryotic single cell RNA MTases. SAH, the side product of the methyl transfer reaction, has been tested on Cmt1 in *Trypanosoma brucei* (196). Further inhibitors derived from the SAH scaffold have been tested on Ecm1 in *E. cuniculi* (197,198), and Cmt1 in *L. infantum* (195).

### Metazoan targets

The organisation of genes and the regulation of their expression increases very significantly when moving from bacteria to single cell eukaryotes and on to metazoans. The human genome codes for hundreds of proteins and at least equally many regulatory RNAs that are involved in shaping the so-called epitranscriptome. Not surprisingly, the human epitranscriptome, dominated by RNA methylations, receives continuously growing attention with respect to its importance for various diseases (199).

RNA methylations, including in particular m^6^A (200–205), m^5^C (206), m^3^C (207) and m^7^G (208–210), were linked to cancer in numerous reports, and their respective MTases proposed as promising drug targets (211). Given their ubiquitous involvement in regulation of gene expression, the implication of RNA methylations in a variety of developmental and metabolic processes is unsurprising, and so is the potential for therapeutic intervention in any number of diseases. Thus, RNA methylations repeatedly emerge in reports on the development of neuronal disorders. For example, the loss of 2′-*O* methylations incorporated by FTSJ1 for example was linked to intellectual deficiencies, and both m^5^C and m^6^A reportedly affect neuronal development and an imbalance of the methylations can lead to malignant phenotypes (212,213). Further prospects for future drug development may be found in human MTases that affect viral replication (214–217), or epigenetic inheritance (218,219).

Mammalian RNA MTases as targets have only recently resurged in the literature. A recent study on SAH analogues with heterocycles in their side chain found activity against the protein methyltransferase PRMT4. At this occasion, the selectivity of the compounds against several other MTases (DNA and RNA) was tested. Remarkably, only one compound (Table 3) showed appreciable activity against the human cap MTase hRNMT with 90% inhibition at a concentration 50 μM. The inhibition in this case was stronger than against the actual targets and might indicate a potential starting point for the development of novel RNA MTase inhibitors (220). In contrast, most of the recent attention in the literature was focussed on the inhibition of *N*^6^ methylation of adenosine (Table 3).

#### METTL3 inhibitors as future therapeutics in cancer treatment

As a consequence of its well documented activity in mRNA methylation and the associated central function in the regulation of gene expression, METTL3, the active subunit in the METTL3–METTL14 complex, has arguably become the best known mammalian RNA MTase, and certainly the most popular as a target for drug development. Being involved in various types of cancer e.g. acute myeloid leukaemia (AML), it has become a central target for new cancer therapies(200–205). Table 3 lists known small-molecule compounds acting on this target, having been detected by methods that cover the complete range from pure *in silico* (166,221) to high end combinations of *in vitro* and *in vivo* techniques (126,222).

Of increased academic interest are the results of an *in silico*-based study, followed up by *in vitro* ³H-SAM methylation assays and cell experiments of selected ligands. Interestingly, several of the latter turned out to activate rather than inhibit the enzymatic activity by a yet unknown mechanism (223).

Another impressive example of a successful application of *in silico* hits towards high affinity METTL3–METTL14 regulators led to the development of spiro molecules from an inhibitor discovered in a virtual screening (221). With the structural adjustments studied, IC_50_ values improved from the low micromolar to the low nanomolar range and furthermore, the compound exhibited m^6^A reduction in cell experiments (224).

**Figure 9.**
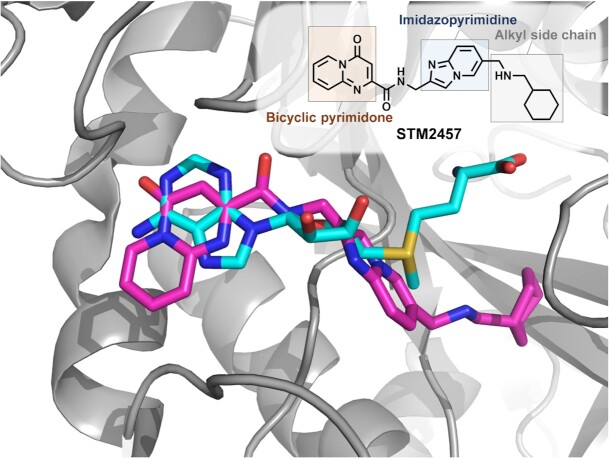
Binding mode of small molecule inhibitors in the catalytic pocket of the METTL3–METTL14 complex. Superimposition of STM2457 (magenta) and SAM (cyan) in complex with METTL3–METTL14. Protein structure created with PyMOL (Schrödinger LLC (2010) The PyMOL Molecular Graphics System, Version 1.3.), PDB IDs used: 7O2I (126), 5L6E (283).

Certainly a milestone was the discovery and characterization of the inhibitor STM2457 (Figure 9) against the METTL3-METTL14 complex (126). The predecessor of this compound emerged from an HTS of 250 000 drug-like compounds and was subsequently optimized for pharmacokinetic and pharmacodynamic properties *in vitro* and *in vivo*. The final inhibitor, as listed in Table 3, exhibited an *in vitro* IC_50_ as low as 16.9 nM.

Conspicuous structural moieties include two heterocycles bearing similarities to purine bases. The terminal bicyclic pyrimidone is linked via an amide to an imidazopyridine. The second heterocycle contains an alkyl side chain consisting of a secondary amine with a terminal cyclohexane ring on the opponent end (Figure 9).

X-ray crystallization showed that the terminal pyrimidone moiety interacts with the adenosyl binding part of the SAM-pocket, whereas the central imidazopyridine occupies the pocket of the transferred methyl group and thereby positioning the terminal alkyl moiety in a different direction from the homocysteine pocket. Due to these two heterocycles the inhibitor still bears analogies to nucleobases without losing its remarkable structural difference from the substrate SAM.

Indeed, STM2457 was developed from one of only two non SAM-like hits in the initial library, yet, despite structural discrepancies of STM2457 compared to SAM, Figure 9 clearly shows that the binding modes of both molecules strongly superimpose in the X-ray structure. Plausibly due to its particular binding mode in the SAM pocket, the compound was found to have remarkable selectivity for METTL3.

#### Transition to antivirals

Many cytostatic drugs used in cancer therapy have functional and structural equivalents among antiviral drugs (225,226). Certain cancer therapy mechanisms include partial or complete shutdown of central metabolic steps, such as DNA replication, transcription, or translation, to deprive fast growing cells of the basis required for their perpetuation, a concept that is also applied to viruses.

By analogy, the concept of targeting human RNA MTases to impede viral replication has been pursued. For example, it has been shown that inhibiting mammalian METTL3 activity depresses viral replication, pointing out new strategies against SARS-CoV-2 or other RNA viruses (216). Further human MTases involved in modification of viral RNA, such as FTSJ3, credibly lend themselves to antiviral research (214).

### Antiviral MTase inhibitors

Many viral pathogens with potential for lasting epi- or pandemics, like the *zika virus* (ZIKV), *dengue virus* (DENV) and most recently the SARS-CoV-2, feature an RNA genome (227–229). Many RNA viruses bring their own RNA MTases coded in their genome. While not all targets and functions of the associated RNA methylations are known, recurrent findings indicate a particular importance of modification of coding RNA, specifically at or near the cap structure. Several cellular antiviral strategies in metazoa aim at identifying viral RNA via its 5′-end structure, or double stranded segments of their genome inside different cellular compartments. Non-canonical 5′-ends, such as a 5′-triphosphate, trigger antiviral pathways that decrease translation and release cytokines. From the widespread occurrence of viral capping enzymes and MTases one can deduce that these manage to mask the presence of viral RNA, at least partially.

Capping starts with an ‘inverse’ attachment of an unmodified guanosine nucleotide via triphosphate bridge, followed by a first methylation at the *N*^7^ of this guanosine. In a second step, the first nucleotide of the RNA sequence is methylated at its ribose 2′-oxygen. While this is performed by distinct catalytic entities, both modification activities reside in the same protein in flaviviruses (230). Given that RNA cap methylations were shown to be crucial for the life cycle, replication, and infection of various virus strains, e.g. flaviviruses and coronaviruses, as well as for escaping the host's innate immune response (231–234), these enzymes represent attractive targets for the development of antiviral compounds (232,235,236). Indeed, this was experimentally underpinned by early work demonstrating that inhibiting viral MTases by SAH, SFG and their derivatives was effective, and that some of the corresponding compounds displayed antiviral activities (150,152,163,237,238).

#### Flaviviral inhibitors

Flaviviruses, with prominent members being *dengue*, *zika* or *west nile virus* (WNV), are the origin of as many as 400 million infections per year, causing serious diseases like encephalitis, hepatitis and fetal death (239). Flaviviruses carry genetic information encoding both *N*^7^ and 2′-*O*-MTase functions in the NS5 protein. This multifunctional protein also contains a guanylyl transferase and an RNA dependent RNA polymerase (RdRp) functionality. Accordingly, the NS5 protein features separate binding sites for its multiple substrates and cofactors. Besides the RNA binding pocket, the SAM binding site is of particular interest for MTase activity, while a GTP binding site is present for the guanylyl transferase part (240–242).

While the *N*^7^ modification is relevant for mRNA translation into viral proteins (243–245), lack of 2′-*O* cap methylation leads to an elevated immune response of the host organism (234,246). Thus, both MTases are considered as drug targets, and several inhibitors against these enzymes have been developed. An overview of these inhibitors is given in Table 4. We will here restrict our discussion to a few examples with particular features.

**Table 4.** List of small molecule inhibitors for viral RNA MTases. The ‘design strategy’ column describes the basic method for inhibitor design, while ‘evaluation method’ roughly lists the experiments conducted in the associated references. CADD = computer aided drug design, i.s. = *in silico*; i.vt. = *in vitro*; i.c. *= in cellulo*; i.vv. = *in vivo*

Organism	Enzyme/modification/RNA species	Design strategy	Evaluation method	References
*dengue virus* (DENV)	NS5/ m^7^G/ mRNA cap	CADD	i.vt.	(301)
			i.s., i.vt.	(302)
			i.vt., i.c.	(303)
		SAH analogue	i.vt.	(248)
			i.vt., i.c.	(304)
		LTS	i.vt.	(305)
		FBDD	i.vt.	(249)
			i.s., i.vt., i.c.	(250)
	NS5/ 2′-*O*/ mRNA cap	CADD	i.vt.	(301)
			i.s., i.vt.	(302,306)
			i.vt., i.c.	(303)
			i.s., i.vt., i.c.	(143)
		Nucleotide analogue	i.vt	(307)
			i.vt.	(308)
			i.s., i.vt., i.c	(309)
		SAH analogue	i.vt., i.c.	(304)
			i.vt.	(248,310)
		LTS	i.vt.	(305)
		FBDD	i.vt.	(249,251)
			i.vt., i.c.	(252)
			i.s., i.vt., i.c.	(250)
	not determined	CADD	i.s.	(311–315,323)
			i.vt., i.c.	(316)
		Nucleotide analogue	i.vt., i.c.	(317)
		LTS	i.s., i.c., i.vv.	(318)
		HTS	i.vt.	(319)
*zika virus* (ZIKV)	NS5/ 2′-*O*/ mRNA cap	Nucleotide analogue	i.s., i.vt.,i.c.	(309)
		FBDD	i.vt.	(251)
			i.s., i.vt., i.c.	(252)
	not determined	CADD	i.s.	(320–322)
			i.s., i.c.	(324)
			i.s., i.vt. i.c.	(316)
		SAH analogue	i.s., i.vt.	(325)
			i.c.	(326)
		HTS	i.s., i.vt.,i.c.	(327)
*west nile virus* (WNV)	NS5/ m^7^G/ mRNA cap	CADD	i.vt.,i.c.	(302)
			i.s., i.vt.,i.c.	(303)
		Nucleotide analogue	i.s., i.vt.,i.c.	(328)
		SAH analogue	i.s., i.vt.,i.c.	(304)
		HTS	i.vt., i.c.	(329)
	NS5/ 2′-*O*/ mRNA cap	CADD	i.vt.,i.c.	(302)
			i.s., i.vt.,i.c.	(303)
		Nucleotide analogue	i.s., i.vt.,i.c.	(328)
		SAH analogue	i.s., i.vt.,i.c.	(304)
		FBDD	i.vt.	(250)
	not determined	Nucleotide analogue	i.s., i.c.	(317)
*yellow fever virus* (YFV)	NS5/ m^7^G/ mRNA cap	CADD	i.vt.	(302,303)
		SAH analogue	i.v., i.c.	(304)
	NS5/ 2′-*O*/ mRNA cap	CADD	i.vt.	(302,303)
		SAH analogue	i.v., i.c.	(304)
		Nucleotide analogue	i.s., i.c.	(309)
	not determined	HTS	i.vt.	(330)
			i.s., i.vt.	(319)
*wesselbron virus* (WV)	NS5/ m^7^G/ mRNA cap	CADD	i.s., i.v.	(301)
	NS5/ 2′-*O*/ mRNA cap	CADD	i.s., i.v.	(301)
*japanese encephalitis virus* (JEV)	not determined	CADD	i.c.	(303)
*St. louis encephalitis virus* (SLEV)	not determined	CADD	i.c.	(303)
*severe acute respiratory syndrome coronavirus* (SARS-CoV)	nsp14/ m^7^G/ mRNA cap	SAH analogue	i.s., i.vt.	(164)
		LTS	i.vt.	(259)
		HTS	i.vt.	(331)
			i.vt., i.c.	(332)
	nsp16/ 2′-*O*/ mRNA cap	LTS	i.vt.	(259)
SARS-CoV-2	nsp14/ m^7^G/ mRNA cap	CADD	i.s.	(267,333–338)
		SAH analogue	i.s., i.vt.	(268,270)
			i.s., i.vt., i.c.	(269)
		HTS	i.vt.	(125)
			i.vt., i.c.	(131,339)
	nsp16/ 2′-*O*/ mRNA cap	CADD	i.s.	(267,337,338, 340–363)
			i.s., i.vt.	(364)
		SAH analogue	i.s., i.vt., i.c.	(269)
		HTS	i.vt.	(365,366)
*chikungunya virus* (CHIKV)	nsp1/ m^7^G/ mRNA cap	GTP analogue	LTP	(367)
		nucleoside analogue	i.c.	(368)
		CADD, nucleoside analogue	i.vt., i.c.	(369,370)
		natural products	i.vt.	(371)
		HTS	i.vt., i.c.	(372)
		HTS	i.c., i.vt.	(373)

A conserved hydrophobic pocket in the SAM binding site of flaviviral NS5 (247), was addressed by designing SAH analogues modified with hydrophobic residues at the *N*^6^ position of the adenine. These compounds were highly potent against (DENV) MTase activity (both *N*^7^ and 2′-*O*) with *K*_i_ values in the sub micromolar range, clearly outperforming SAH. Furthermore, some selectivity towards human MTases was observed (248).

Against the same DENV MTase, a fragment-based screening was performed using a thermal shift assay (TSA). 500 drug-like fragments were screened of which 32 fragments were retained. Five fragments were able to inhibit at least one of the MTase activities, while seven of these hits could be co-crystallized with the enzyme. The fragments bound to four distinct sites, one corresponding to the GTP binding pocket of the enzymes guanylyl transferase functionality. Interestingly, the other fragments bound to three previously unexplored binding sites. Two of these were in close proximity to each other and to the SAM binding site (249).

Fragments found in these pockets were linked together based on the crystal structures and submitted to further fragment growing (*vide supra*) to fill out their respective binding sites, without reaching into the SAM binding site. This led to compounds which did not affect the *N*^7^ MTase function, but exclusively inhibited the 2′-*O* function. IC_50_ values were in the 3-digit micromolar range, with the best compound displaying an IC_50_ value of around 90 μM. The newly designed inhibitors were also able to reduce *west nile virus* and *zika virus* MTase activity with IC_50_ values in the same order of magnitude (250,251).

**Figure 10.**
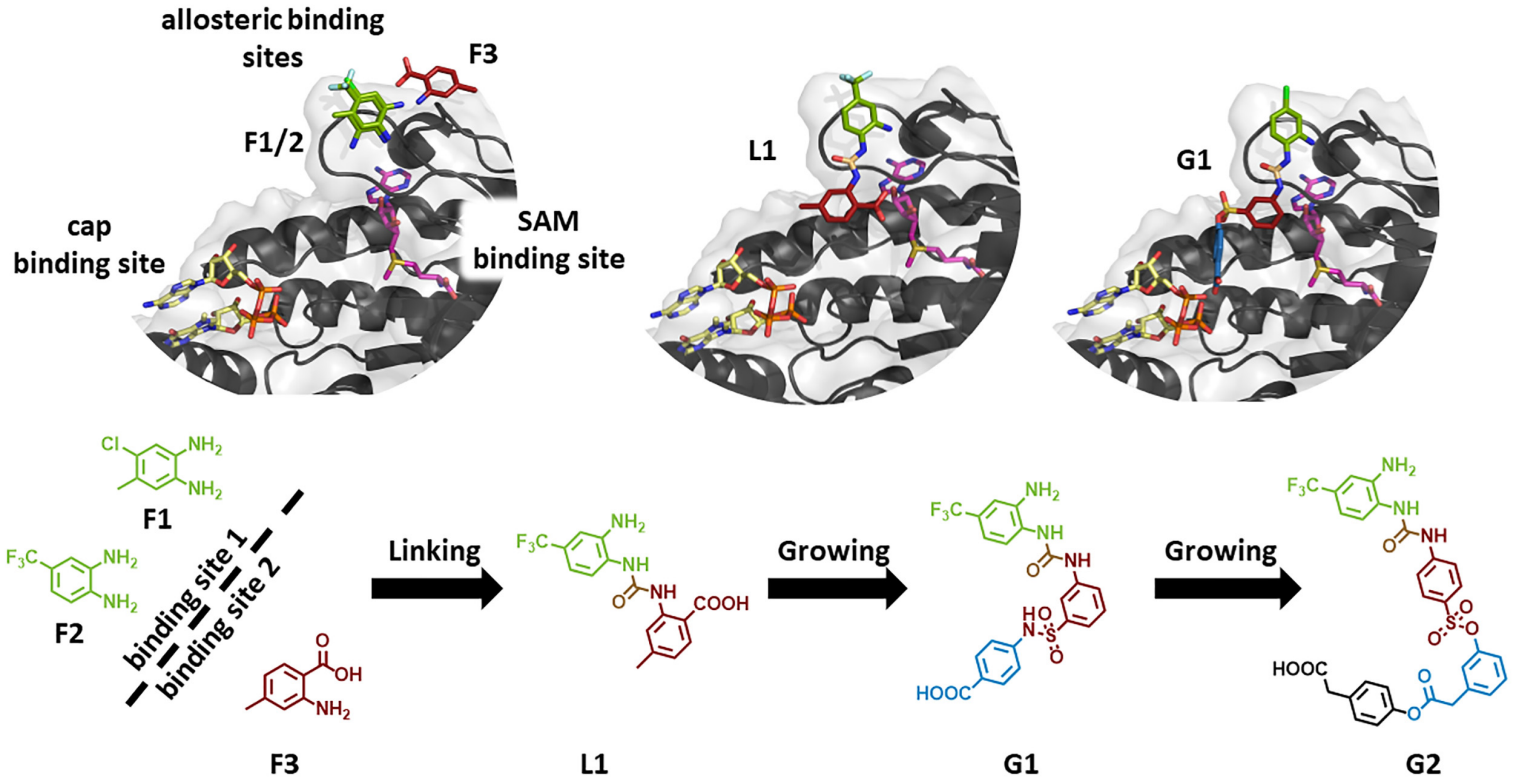
Fragment-based drug design (FBDD) of allosteric inhibitors for flaviviral 2′-*O* MTases. IC_50_ values range from single digit millimolar range for fragments F1, F2 and F3 over around 90 μM (G1) to 24 μM (G2). Figure adapted from Hernandez *et al.* (252). Protein structures were created with PyMOL (Schrödinger LLC (2010) The PyMOL Molecular Graphics System, Version 1.3.). PDB IDs used: 5EKX, 5EIW, 5EIF, 5EHG, 5E9Q, 5EHI (250), 5WZ2 (284).

These allegedly allosteric inhibitors were further optimized using an *in silico* pipeline, in which a focused library was created around the essential scaffold, and subsequently docked to the DENV MTase. This led to compounds inhibiting DENV and ZIKV 2′-*O* MTase activity, with the best candidates having IC_50_ values around 20 μM. None of the compounds displayed antiviral activity (252) (Figure 10).

#### Coronaviral inhibitors

The RNA based genome of coronaviruses has been repeatedly scrutinized for antiviral targets in the course of meanwhile three major epi- and pandemics since the start of the century (253). These activities have picked up considerable momentum with the onset of the latest pandemic in late 2019, and, not surprisingly, they also focused attention on the two MTase activities of SARS-CoV-2 encoded proteins.

In coronaviruses the *N*^7^ MTase function resides in nsp14. MTase dysfunction was not only associated with decreased viral replication and translation, but also with reduced ability of the virus to shut down hosts translation and innate immune response (254–257).

The 2′-*O*-MTase function is located on nsp16 which needs to form a complex with nsp10 to be active (258–264). As in flaviviruses, the 2′-*O*-methylation contributes to evasion of the host's immune response, making both MTases suitable drug targets (265,266). An overview over the meanwhile vast literature on inhibitors against coronaviruses is given in Table 4. We here outline selected examples of special interest from the different approaches used.

Remarkable is the high number of computational studies published in the short time period since the breakout of SARS-CoV-2. As a case in point, an *in silico* study implemented an ultra large virtual screening against all relevant viral proteins. Correspondingly, suggested potential inhibitors included such directed against both MTases (267).

Of course, druggability of coronaviral MTases was not only a topic of *in silico* studies, but also of a sizable number of wet lab campaigns. Both SARS-CoV-2 MTase functions, i.e. nsp14 and 16, respond to inhibition by SAH analogues. Remarkably, some of the latter reached IC_50_ values in the nanomolar range (268–270). The more sophisticated concept of SAH-like bisubstrate inhibitors, targeting both, the SAM and RNA binding sites (*vide supra*), was also applied to inhibition of SARS-CoV and SARS-CoV-2 MTases (164,270).

**Figure 11.**
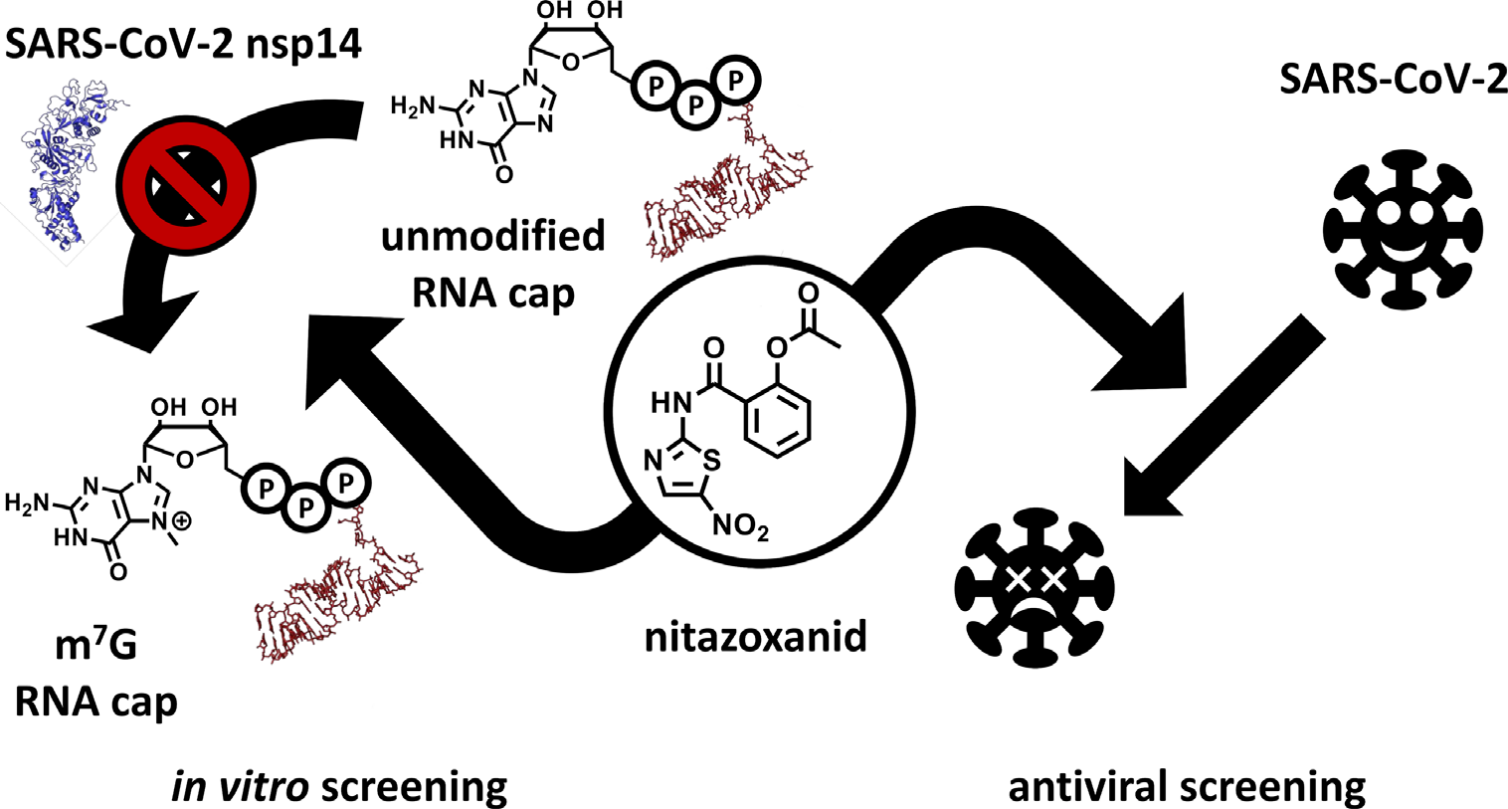
Drug repurposing against SARS-CoV-2. Nitazoxanid was identified in an *in silico* screening against nsp14 and independently as a hit in an antiviral screening against SARS-CoV-2 (125) and is now tested in clinical trials (NCT04486313, NCT04459286, NCT04348409). Protein and RNA structures were created with PyMOL (Schrödinger LLC (2010) The PyMOL Molecular Graphics System, Version 1.3.). PDB ID used: 7N0B (285).

A serendipitous MTase inhibitor discovery resulted from a screening of a library of 1771 FDA approved drugs for repurposing against SARS-CoV-2. Among 12 hits against nsp14, nitazoxanide provided the most compelling effects. With an IC_50_ value of around 10 μM it displayed only moderate affinity but was selective for nsp14 when compared with hRNMT. Importantly, this compound was previously shown to inhibit SARS-CoV-2 activity in cell culture (125) and is currently being investigated in clinical studies against coronavirus disease 2019 (COVID-19) some of which are concluded (Figure 11, NCT04486313, NCT04459286, NCT04348409, NCT04746183, as of 02/2022).

### Macromolecular approaches to RNA MTase inhibition

In addition to classical approaches using small molecule inhibitors, experimental approaches based on macromolecules are being put forward at an increasing rate. As a recent example, disruption of the coronavirus nsp10-nsp16 complex was achieved using peptides of around 1.3 to 4.9 kDa, thereby abolishing enzymatic activity (271,272).

Aptamers are structured RNAs whose specific binding to targets can be directed during their genesis in an *in vitro* selection process called SELEX (systematic evolution of ligands by exponential enrichment) (273,274). Such RNAs have been employed in diverse roles in drug discovery, including competitive binding to targets. Aptamers were developed against the MTases from DENV and *japanese encephalitis virus* (JEV). The best RNAs were found to bind in the low nanomolar range and inhibited the *N*^7^ and 2′-*O* MTase functions. In cells, which were co-transfected with the JEV genome and an aptamer against JEV, replication of the viral RNA was impeded pointing to a potential antiviral activity of the aptamer (273,274).

**Figure 12.**
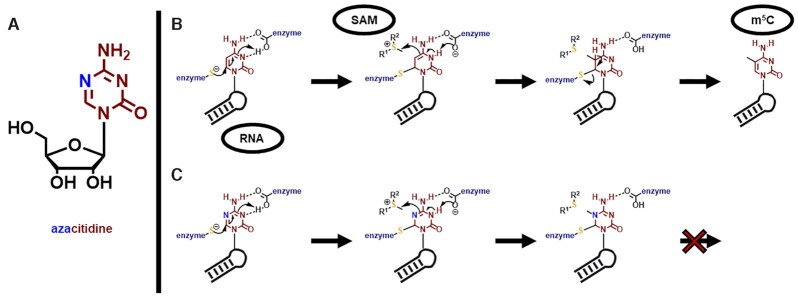
Azacitidine mode of action. (**A**) Molecular structure of azacitidine. (**B**) General mechanism for implementation of m^5^C on RNA for m^5^C RNA methyltransferases. (**C**) Mechanism of action of azacitidine modified RNA for m^5^C RNA methyltransferases in which the enzyme dissociation cannot take place.

A special case in the field of macromolecular RNA MTase inhibitors is azacitidine, a ribonucleoside drug used for the treatment of leukemia and myelodysplastic syndromes (Figure 12A). Chemically it belongs to the group of cytidine analogues and is incorporated into RNA and DNA upon uptake into cells. As suicide bait, it is able to trap the corresponding MTases with its electron deficient aromatic ring on the nucleic acid and thereby inactivating them as illustrated in Figure 12B and C (275,276). Azacitidine was shown to inhibit the human RNA MTases Dnmt2 and Nsun2, both m^5^C MTases (277–279).

### Highlights and shortcomings in medicinal chemistry of RNA MTases

The development of inhibitors of RNA dependent MTases features a long, but slow history, that has only recently accelerated, albeit now with sustained motivation and impact. Will the incentive of working in a field, which has suddenly become ‘timely’, lead to new concepts and approaches in medicinal chemistry, and will there be impact beyond academia? There is an obvious and direct need for potent hits, leads, and drug-like inhibitors as antibiotics, antivirals, and in cancer therapy. Aided by a general hype in this newly minted field of epitranscriptomics, venture capital in the order of 10^9^ $ was recently raised and invested into biotech startups (8–11). Unfortunately, commercially driven target selection still follows the usual preferences. Thus, before the advent of SARS-CoV-2 in our midst, cancer therapy was largely predominant, and likely will be again.

Upheld only by academia and a token commitment of Big Pharma, antibiotics research aimed at overcoming strains with multiple resistance is badly needed, but apparently lacking sufficient financial incentive. A case in point is the ErmC family of bacterial MTases, a long-standing prime study object for learning important lessons. Its methylation activity on the bacterial ribosome, which confers multiple resistances (180), is orthogonal to any MTase in humans, making specificity a minor problem; yet clinical trials are not in sight. In contrast, human METTL3 activity has come to the limelight only very recently, and although drugging this human enzyme must face scrutiny with respect to cross inhibition of a score of other human MTases, lead development was impressively fast (126). Here we see the importance of lessons learned from other domains of medicinal chemistry, which taught us that even within a large group of enzymes employing the same cofactor (e.g. kinases (3–5)), compounds may be developed that combine efficient inhibition with high specificity.

Thus, at least in academia, the stage is set for a boost in RNA MTase related developments in medicinal chemistry. The field urgently needs better assays for medium and high-throughput screening efforts. Inhibitors need to be applied as tools in fundamental research, involving cell biologists early in the game. Fundamental insights on specificity, selectivity, and bioavailability of competitive inhibitors, such as SAM analogues, are missing, as are – with few exceptions – allosteric inhibitors. Our understanding of escape strategies in antibiotic, anticancer and antiviral settings is still in its infancy.

Again, funding will have a decisive impact, but in contrast to past decades, it will be more easily allocated to this topic. Sad though it is, editors of high impact journals are also more likely to consider sending manuscripts out for review, if the topic is ‘timely’, and for a while still, RNA methylation will be. On the downside, the epitranscriptomic field is currently facing a flood of superficial copycat papers, whose character is likely to eventually spill over into the medchem field.

Many of the above facets, including negative ones, have recently been developed in fast forward time lapse. The thus far ultimate demonstration of what a joint worldwide effort can achieve in the field of MTases is arguably the development of inhibitors against SARS-CoV-2 MTases in the context of the pandemic. Its volume can be gleaned from the entries in Table 4, and although drug development is a matter of decades rather than years, serendipity originating from a repurposing approach has it, that an inhibitor of a SARS-CoV-2 RNA MTase is now in clinical trials (125).

## GLOSSARY

**β,γ-unsaturated chain** double- or triple-bond, seperated by one carbon atom from a specific position (e.g. functional groups, SAM scaffold or RNA molecules, Figures 2B and 3A).

**backboding/** **π-backbonding** stabilization of chemical bonds by additional electron donation via → *π-orbitals* (Figure 3A).

**CADD** computer aided drug design/discovery, drug-design method based on or supplemented with → *in silico* methods, such as molecular docking.

**chalcogen** element of the 6^th^ main group in the periodic table (e.g. oxygen, sulfur, selenium, tellurium).

**chirality** molecules which cannot be overlaid with their mirror images by rotation, as the left and right hand. The two different isomer forms are dubbed *R*- and *S*-isomers.

**click chemistry** various reaction types used for bioconjugation. Relevant reactions are displayed in Figure 6A.

**double activated SAM analogues** SAM analogues containing → *β,γ-unsaturated side chains* used in → *mTAG* RNA labelling (Figures 2B and 3A).

**epitranscriptomics** research centered around RNA modifications.

**FBDD** fragment-based drug design/discovery, drug-design method in which small molecular fragments are first screened and then rationally improved towards their respective targets.

**HTS** high throughput screening, drug-design method in which thousands of molecules are screened towards their respective targets to identify novel enzyme inhibitors.

***in silico*** experiments conducted on a computer.

***in situ*** emerging of the molecule in question directly ‘on site’, i.e. in the reaction mixture.

**IC_50_** half maximal inhibitory concentration, a widely used value for quantifying the potential of inhibitiors.

***K*_i_** equilibrium constant describing binding affinity of the enzyme–inhibitor complex as calculated by the Cheng–Prussov equation, a widely used value for quantifying the potential of inhibitiors.

**mTAG** methyltransferase-directed transfer of activated groups, labelling technique for biomolecules exploiting promiscuous MTases and → *double activated SAM analogues* (Figure 2B).

***N-*mustard derivatives** compounds used for → *SMILing* which generate the corresponding aziridine derivatives → *in situ* as displayed in Figure 2.

**orthogonal click reactions** → *click reactions*, that can independently and selectively address different functional groups and thus allow multiple labelling.

**π-orbital** oribtals responsible for double- and triple-bonds located above the binding plane (Figure 3A).

**racemate/racemic mixture** an even mixture of corresponding *R*- and *S*-isomers of the same, → *chiral* molecule (Figure 3B).

**SAR** structure–activity relationship, correlation between the molecular structure of a compound and its biological activity.

**SMILing** sequence-specific methyltransferase-induced labelling, labelling technique for biomolecules exploiting promiscuous MTases and aziridine derivatives of SAM (Figure 2A).

**sp^2^ hybridisation** electron distribution as found in double bonds leading to their planar shape. In carbon atoms three sp^2^-hybrid orbitals exist together with one p-orbital.

## DATA AVAILABILITY

All relevant data are available in the manuscript itself.
